# Hydrochemical and GIS-based evaluation of groundwater suitability for irrigation using IWQI in the desert hinterland of western Nile Delta Egypt

**DOI:** 10.1038/s41598-026-39089-z

**Published:** 2026-03-10

**Authors:** Youssef A. Youssef, Mohamed E. Abuarab, Ahmed Mahrous, Eslam Farag, Liu Yan-Li, Yan Wen-Hui, Alaa M. Kasem, Abd Al-Rahman S. Ahmed

**Affiliations:** 1 Agricultural Engineering Department, Faculty of Agriculture, Cairo University, Giza, 12613 Egypt; 2 Agriculture Applications Department, National Authority for Remote Sensing and Space Sciences, Cairo, Egypt; 3 Department of Hydrology and Water Resources, Nanjing Hydraulic Research Institute (NHRI), Nanjing, PR China; 4 Department of Natural Resources, Faculty of African Postgraduate Studies, Cairo University, Giza, 12613 Egypt

**Keywords:** Groundwater quality, Irrigation water quality index, Hydrochemical assessment, GIS spatial mapping, Sodicity and salinity hazards, Environmental sciences, Hydrology

## Abstract

Groundwater is an essential resource for irrigation in the newly reclaimed regions of the New Delta in Egypt, where the sustainable advancement of agriculture heavily depends on its quality. This study aimed to assess the suitability of the water for both drinking and irrigation purposes in the western hinterland of the Nile Delta, and to aid water resource managers and policymakers in recognizing the potential risks linked to the utilization of this water. A total of 41 groundwater samples were analyzed for major cations and anions (Ca²⁺, Mg²⁺, Na⁺, K⁺, HCO₃⁻, Cl⁻, SO₄²⁻), and key irrigation indices, such as SAR, Na%, RSC, PI, KR, MAR, and IWQI were calculated. The primary findings revealed that the Irrigation Water Quality Index (IWQI) classified 7.32% of the samples as having moderate restrictions, 34.15% as having high restrictions, and 58.54% as having severe restrictions, underscoring the widespread issues of salinity and sodium hazards across different zones. The results indicated that most samples fall within acceptable to permissible limits; however, high levels of Na⁺ and Cl⁻ in certain areas suggest potential challenges related to sodicity and salinity. Spatial distribution maps confirmed that these risks are localized and closely linked to soil texture and irrigation practices. Principal Component Analysis (PCA) illustrated that the first two components account for nearly 70% of the total variance, with salinity-related variables (EC, TDS, Na⁺, Cl⁻, SAR) being the dominant factors, followed by sodium–chloride enrichment, carbonate equilibria (HCO₃⁻, RSC, pH), and evaporation effects. In conclusion, the findings highlight that while a considerable portion of the groundwater is still suitable for irrigation, ongoing use without proper management could result in decreased permeability and heightened soil sodicity. Therefore, it is advisable to adopt adaptive management strategies, including crop selection, blending of water sources, and regular monitoring.

## Introduction

 All living organisms rely on water as one of their most essential natural resources. This resource is particularly significant for sustainable economic development in arid regions. The challenges posed by urbanization, human activities, and an increasing population exacerbate this situation^1,2^. By the year 2030, it is projected that approximately 40% of the global population will experience the repercussions of water scarcity^3,4^. Consequently, groundwater is crucial for fulfilling human needs and sustaining life; thus, global management of water resources is imperative^5–7^. This underscores the necessity for thorough evaluation and management of this vital resource.

Groundwater stands as one of the most critical water resources for humanity. The demand for water continues to escalate, while surface water supplies are diminishing, particularly in arid and semi-arid areas. This water serves as the primary source for drinking and agricultural purposes in numerous countries across the globe. Globally, groundwater extraction ranges from 750 to 1,500 km³ each year^8–10^, supporting over 2 billion individuals^11^. It accounts for 50% of drinking water, 33% of industrial water, and 40% of agricultural water usage worldwide^12^.

In Egypt, over 85% of the water budget is allocated for agricultural activities^13^. The country has recently faced an increased demand for water due to its expanding population, new significant land reclamation initiatives, and industrial growth. To satisfy agricultural requirements, these initiatives predominantly depend on surface water, although there has been a recent shift towards utilizing groundwater^1,14^.

Water quality remains a significant issue worldwide, leading governments to seek additional water resources to satisfy the needs of industrial, agricultural, and residential sectors. According to UNESCO’s 2021 World Water Development Report^15,16^, global freshwater consumption has surged six-fold over the last century, with a steady annual growth rate of approximately 1% since the 1980s. Furthermore, in recent decades, water shortages caused by unregulated groundwater resource exploitation have adversely affected both groundwater quality and levels^17–20^.

Groundwater serves as a crucial resource for drinking and agriculture. Monitoring its quality is vital to ensure sustainable usage. This evaluation analyzes physical and chemical characteristics and benchmarks them against global standards set by the World Health Organization (WHO) and the Food and Agriculture Organization (FAO). This procedure aids in assessing the water’s suitability and mitigating health and agricultural risks associated with it^21,22^.

Geographic Information Systems (GIS) and remote sensing (RS) have become essential tools for the management and evaluation of water resources, particularly groundwater^23,24^. Satellite imagery and spatial data sources enable us to monitor alterations in water quality caused by land use, urbanization, agricultural practices, and climate change^25,26^. Remote sensing methods, such as Landsat 8, are commonly employed to assess water quality by identifying parameters like turbidity and chlorophyll levels in aquatic environments^27,28^. GIS effectively amalgamates geographical, chemical, and field data to create maps that depict the distribution of water quality^29^. As noted by Quinn et al., these maps are instrumental in pinpointing areas that are most susceptible to degradation and offer guidance for sustainable water management practices^30,31^. The combination of GIS and RS has proven to be highly effective in assessing and delivering comprehensive insights into vital aspects such as water quality, groundwater management, and soil moisture^32–34^. By merging these spatial tools with traditional on-site sampling methods, we can achieve a more thorough and precise evaluation of water quality across diverse water systems. This approach enhances our understanding of the spatial patterns and changes impacting water resources^35,36^.

The Water Quality Index (WQI) serves as a prevalent instrument for assessing and categorizing groundwater. It translates a collection of physical and chemical measurements into a singular digital value that clearly indicates quality levels^37,38^. The WQI evaluates the concentration of various chemical constituents, including total dissolved solids (TDS), pH, primary ions (Ca²⁺, Mg²⁺, Na⁺, Cl⁻, SO₄²⁻, HCO₃⁻), and trace elements, against the acceptable thresholds established by the World Health Organization (WHO) for potable water^39–42^. Depending on the indicator’s value, water is categorized as excellent, good, medium, weak, or unsuitable^43,44^. The significance of the WQI lies in its capacity to distill complex analyses into a format that offers decision-makers and researchers a lucid comprehension of the safety of groundwater for human consumption.

Given that numerous indicators have been established to assess their appropriateness for agricultural applications, the quality of groundwater for irrigation is a vital factor in achieving sustainable agricultural productivity^45^. High sodium concentrations can lead to soil dispersion and a diminished capacity for infiltration over time; thus, the sodium absorption ratio (SAR) is among the most crucial and widely utilized indicators for assessing the impact of sodium on soil permeability and structure^46–49^. Key indicators for evaluating the ionic balance and potential risks in the soil include the percentage of sodium (NA%), the residual sodium carbonate (RSC), the magnesium ratio (MH), and the Kelly ratio (KR)^50–52^. Additionally, the long-term impacts of irrigation water are anticipated in terms of altering the soil’s capacity to convey water or the hydric properties of the soil using the permeability index (PI)^53,54^.

The irrigation water quality index (IWQI) was developed as a holistic instrument aimed at enhancing precision by amalgamating various indicators, including SAR, NA%, RSC, MH, KR, and PI, into a singular numerical representation. This facilitates a comprehensive and unbiased evaluation of the extent to which water is suitable for irrigation^53,55^. The integration of these indicators offers a nuanced understanding of the potential effects of irrigation water on crop yield, soil vitality, and the sustainability of agricultural practices^56,57^.

The aims of this study were to: (I) Evaluate the quality of groundwater in the desert hinterland of the Nile Delta, which encompasses three governorates, specifically EL-Giza, AL-Beheira, and Alexandria, (II) compute the irrigation water quality index in these regions to determine the areas that are appropriate for agricultural development projects based on groundwater levels, quality, and suitability for irrigated crops, (III) identify potential sources of pollution and assess their effects on agricultural productivity and public health, and (IV) engage with stakeholders and decision-makers to ensure that the findings of the research can be utilized to guide agricultural investments towards the most suitable regions according to the irrigation water quality index, thereby optimizing groundwater use and achieving water and food security in the region.

## Materials and methods

### Study area

**Fig. 1:**
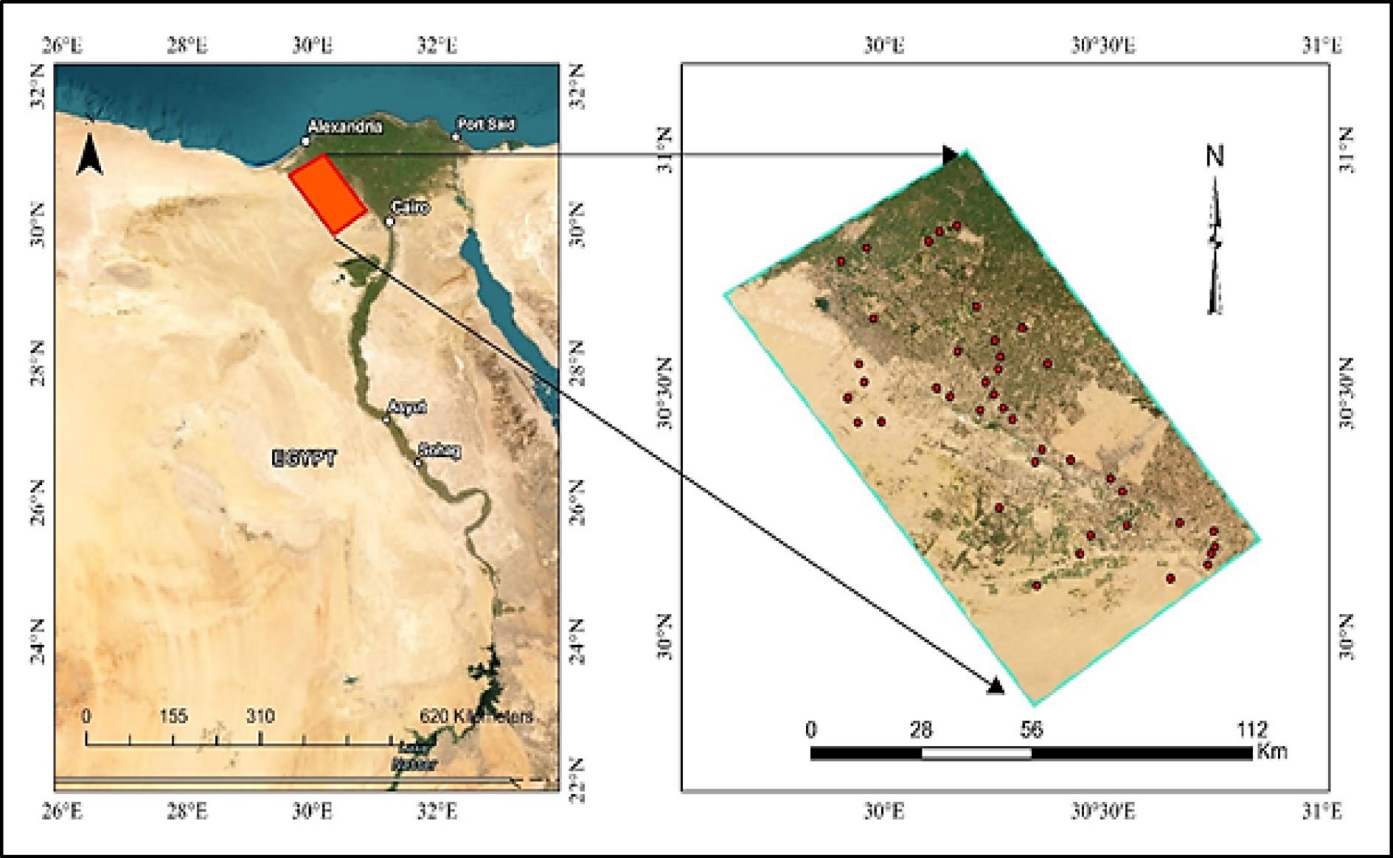
Location map of the groundwater wells and the study area.

The study area is located in the western Nile Delta of Egypt. This region is recognized as one of the most fertile agricultural zones in the country (Fig. 1), which was created using ArcGIS Pro 3.0.2. Covering an area of over 7486.83 km², it lies between latitudes 29°51’14” – 31°0’11” N and longitudes 29°37’49” – 30°51’45” E, bordered to the east by the Rosetta branch of the Nile and gradually extending westward into the desert. The elevation typically ranges from 5 to 25 m above sea level, and the landscape is predominantly flat with gentle slopes.

The climate in this region is classified as arid to semi-arid^58^, characterized by minimal and unpredictable rainfall, which mainly occurs in the winter months. While the annual evapotranspiration demand exceeds 1500 mm^59^, the average yearly precipitation is generally less than 200–250 mm^60^. This significant discrepancy leads to a heightened dependence on groundwater resources. Summers are typically dry, while winters are warm, with rainfall occurring primarily along the coast.

The predominant use of land in this area is for agricultural purposes, which encompasses a diverse range of crops such as cereals, vegetables, and fruit trees, all of which benefit from comprehensive irrigation systems. The expansion of cultivated land, coupled with the use of chemical pesticides and fertilizers, raises significant concerns about the sustainability of groundwater quality. These conditions underscore the importance of ongoing monitoring and evaluation of the water’s appropriateness for irrigation within this delicate ecosystem.

The groundwater system of the study area comprises the Quaternary Nile Delta aquifer and the underlying Lower Miocene (Moghra) aquifer. The Quaternary aquifer consists of unconsolidated Pleistocene–Holocene alluvial deposits, mainly sands and gravels with interbedded clay layers. The Moghra aquifer is composed predominantly of fluvial sands and gravels with minor clay intercalations and occurs under unconfined to semi-confined conditions^61,62^.

The two aquifers are hydraulically connected, allowing vertical and lateral groundwater exchange. Regional groundwater flow is directed westward along a declining hydraulic gradient from near sea level in the eastern Delta toward approximately − 40 m in the western parts, with local flow patterns strongly modified by intensive groundwater abstraction^63–67^. Groundwater evolution along this flow path is characterized by progressive water–rock interaction, resulting in the dissolution of carbonate, silicate, and sulfate minerals, while evaporation and mixing with deeper saline groundwater and marine-influenced sediments contribute to increased salinity in down gradient and northern parts of the aquifer system.

### Collection of groundwater samples

A total of 41 groundwater samples were collected from both production and monitoring well after purging the boreholes by continuous pumping for approximately 15 min to remove stagnant water. All samples were collected from private farms. Permission was obtained from the owners of these farms, with the clarification that these results will be published through scientific research and publications, and that this research aims primarily to monitor the current state of groundwater in order to take the necessary measures in the future towards the sustainable management of groundwater as the main source of irrigation in this region. The water samples were gathered in sterile plastic containers for subsequent physicochemical analysis.

Following the guidelines set by APHA, the samples were analyzed for various parameters at the National Research Center laboratory^68,69^. The physicochemical analysis included several key parameters such as pH, electrical conductivity (EC), and total dissolved solids (TDS). Additionally, soluble cations like calcium (Ca^2+^), magnesium (Mg^2+^), sodium (Na⁺), and potassium (K^+^) were measured, along with soluble anions such as chloride (Cl^−^), sulfate (SO_4_^2−^), bicarbonate (HCO_3_^−^), and carbonate (CO_3_). The pH levels were determined using a glass electrode pH meter. A flame photometer was employed to assess the concentrations of sodium and potassium, while calcium and magnesium were titrated using EDTA. Chloride concentrations were measured using Mohr’s method, and the titration of carbonate (CO_3_) and bicarbonate (HCO_3_^−^) was conducted with H_2_SO_4_ in the presence of phenolphthalein and methyl orange indicators, respectively. Sulfate estimation was performed by precipitation with barium chloride. Electrical conductivity was measured using an EC meter in a 1:1 extract. The physicochemical characteristics and statistical data of the groundwater samples collected from the study region have been compiled. The parameters analyzed include pH, EC, TDS, Ca^2+^, Mg^2+^, Na^+^, K^+^, HCO_3_^−^, Cl^−^, and SO_4_^2−^).

### Irrigation water quality

The quality of irrigation water was assessed using several critical indices, which were derived from Equations presented in Table 1. These indices include the Permeability Index (PI), Magnesium Adsorption Ratio (MR), Sodium Adsorption Ratio (SAR), Residual Sodium Carbonate (RSC), Kelley Ratio (KR), and sodium percentage (NA%). Utilizing the hydrochemical data obtained, we calculated these indices. The standards for groundwater quality parameters (pH, TDS, Na⁺, HCO₃⁻, Cl⁻, and SO₄²) were established alongside irrigation indicators (PI, MR, SAR, RSC, KR, and NA%) (Table 2). According to irrigation guidelines, water is deemed unsuitable if the SAR exceeds 10 meq/L and the sodium percentage is greater than 80%^49,70–72^.

**Table 1:** Determined the water quality parameter using the parameters that were measured.

Quality parameter	Unit	Formula applied	Reference
Sodium adsorption ratio	(Meq L^− 1^)	$\:SAR=\frac{\mathrm{N}\mathrm{a}\:}{\sqrt{\frac{Ca+Mg}{2}}}$	^80^
Magnesium adsorption ratio	(%)	$\:MR=\frac{Mg}{Mg+Ca}*100$	^81^
Percentage of sodium	(%)	$\:{N}_{a}=\frac{Na+K}{Ca+Mg+Na+K}*100$	^82^
Permeability index	(%)	$\:PI=\frac{\mathrm{N}\mathrm{a}+\sqrt{{HCO}_{3}}}{Ca+Mg+Na}*100$	^83,84^
Residual sodium carbonate	(Meq L^− 1^)	$\:RSC=({HCO}_{3}+{CO}_{3})-(\mathrm{M}\mathrm{g}+\:\mathrm{C}\mathrm{a})$	^85^
Kelly ratio	(Meq L^− 1^)	$\:KR=\frac{Na}{Ca+Mg}$	^73^

**Table 2:** The standards of groundwater quality parameters and irrigation suitability indices.

Parameters	Class	Range	Notes	References
pH	Excellent	6.5–8.4	Optimal range for irrigation	^49,86^
	Unsuitable	< 6.5 or > 8.5	Can harm soil and crops	^49^
TDS (mg L^− 1^)	Excellent	< 450	Excellent quality for irrigation	^49,86^
	Good	450–2000	Suitable for most crops	^49^
	Doubtful	2000–3000	Requires salt-tolerant crops	
	Unsuitable	> 3000	Not suitable for irrigation	
Cl⁻ (meq L^− 1^)	Excellent	< 4	No restriction	^49^
	Good	4–10	Slight to moderate restriction	
	Unsuitable	> 10	Severe restriction	
Na⁺ (meq L^− 1^)	Excellent	< 3	No restriction	^49^ ^86^
	Good	3–9	Moderate restriction	
	Unsuitable	> 9	Severe restriction	
SO₄²⁻ (meq L^− 1^)	Excellent	< 5	No restriction	^49^
	Good	5–20	Moderate restriction	
	Unsuitable	> 20	Severe restriction	
HCO₃⁻ (meq L^− 1^)	Excellent	< 1.5	No restriction	^49^ ^49,87^
	Good	1.5–8.5	Moderate restriction	
	Unsuitable	> 8.5	Severe restriction	
SAR	Excellent	< 10	Excellent quality	^49^
	Good	10–18	Moderate restriction	
	Doubtful	18–26	Requires management practices	^49^ ^87^
	Unsuitable	> 26	Not suitable	
RSC (meq L^− 1^)	Excellent	< 1.25	No restriction	^52^
	Good	1.25–2.5	Moderate restriction	^49^
	Unsuitable	> 2.5	Severe restriction	^52^
KR (meq L^− 1^)	Excellent	KR < 1	Water is safe for irrigation, no problem from sodium	^49^
	Unsuitable	KR > 1	Sodium control over other cations, causing problems for the soil	^49^ ^88^
Na (%)	Excellent	< 20%	Excellent	^88^ ^76^
	Good	20–40%	Good	
	Permissible	40–60%	Permissible	
	Doubtful	60–80%	Doubtful	
	Unsuitable	> 80%	Unsuitable	
MR	Excellent	< 50%	Suitable	^76^
	Unsuitable	> 50%	Unsuitable	
PI	Excellent	> 75%	Excellent	^72,77,78^
	Good	25–75%	Good	
	Unsuitable	< 25%	Unsuitable	

To ensure the precision of the chemical analyses, Eq. (1) was employed to determine the ion charge balance error. Moneam^73^ indicated that variations should remain within ± 10%, and the findings exhibited sufficient accuracy.

1 $$Error{\text{ }}of{\text{ }}ion{\text{ }}ch\arg e{\text{ }}balance{\text{ }} = \frac{{\sum Cations - \sum Anions}}{{\sum Cations + \sum Anions}} \times 100$$

Where ∑Cations is the total of the cations (Ca^2+^, Mg^2+^, Na^+^, and K^+^), measured in meq L^− 1^, and ∑Anions is the total of the anions (CO_3_, HCO_3_^−^, Cl^−^, and SO_4_^2−^), also measured in meq L^− 1^.

The importance of magnesium for soil structure and plant development is illustrated by the MR index. An MR value below 50 is deemed advantageous. Due to the additional magnesium, elevated levels may adversely affect calcium absorption^74–76^. The concentrations of carbonates and bicarbonates are analyzed through the RSC parameter. Soil fertility may suffer from the precipitation of calcium and magnesium when concentrations exceed 1.5 meq L^− 1^^74–76^.

The irrigation water is categorized into three groups based on the PI index. In general, Class I and II waters, with PI values above 75% and between 25 and 75%, are considered suitable for irrigation. In contrast, water with PI values below 25% (Class III) is regarded as low quality and inappropriate for agricultural purposes^72,77,78^. The Kelley ratio (KR) is utilized to evaluate the proportion of sodium to total calcium and magnesium in water (expressed in meq L^− 1^). A KR value of less than 1 indicates that the water is suitable, as higher values imply that soil characteristics may influence permeability. Recent research, such as that by Nkpoidet, Udosen^79^, has also employed this method to determine the suitability of water for irrigation.

### Spatial distribution maps

To guarantee spatial precision, the sampling sites were initially documented in the field with a handheld Global Positioning System (GPS), and the gathered coordinates were later imported into a Geographic Information System (GIS) database for additional analysis. The application of GIS offers a strong framework for merging water quality data with their spatial context, thus facilitating a more comprehensive understanding of hydrochemical variations throughout the study area. The spatial distribution of groundwater quality parameters was examined utilizing ArcGIS Pro (version 3.0.2).

Groundwater quality maps were generated in a GIS environment using inverse distance weighting (IDW), a deterministic interpolation method that estimates values at un-sampled locations based on distance-weighted averages of nearby observations. Although Kriging can yield optimal estimates by modelling spatial autocorrelation, but it requires sufficient and evenly distributed samples for stable variogram estimation. When sampling is sparse or irregular, deterministic methods such as IDW are a practical alternative for mapping spatial patterns of environmental variables^89,90^. Given the limited and irregular sampling distribution in the present dataset, variogram estimation was not robust; therefore, IDW was selected for mapping groundwater quality patterns. The inverse distance weighting (IDW) method is particularly valued for its computational efficiency and effectiveness when it comes to generating groundwater quality maps and managing water resources, especially in comparison to kriging, which is considered a more advanced and sophisticated geostatistical methodology that may require a greater level of data availability. In situations where data is sparse, particularly in less populated or rural areas, IDW is frequently employed for its practicality and has found common application in various agricultural research settings, as highlighted in studies conducted by Tayyab, Aslam^91,92^.

The Model Builder application within the ArcGIS framework was instrumental in the development of a sophisticated spatial decision model, which served the purpose of delineating zones that are suitable for groundwater-based irrigation. The initial phase of this analytical process involved the integration of essential hydrochemical raster datasets—specifically, parameters such as Percent Irrigation (PI), Residual Sodium Carbonate (RSC), Sodium Adsorption Ratio (SAR), Potassium Ratio (KR), Sodium Percentage (Na%), and Total Dissolved Solids (TDS)—and subsequently standardizing these variables onto a singular integer scale that ranges from 1 to 9, where a higher value indicates an increased level of suitability for irrigation practices. Following this initial reclassification, all the reclassified raster layers were processed through the Weighted Overlay tool, wherein specific percentage-based weights were allocated to each parameter (TDS receiving 35%, SAR 20%, Na% 20%, RSC 15%, PI 5%, and KR 5%), all in an effort to derive a comprehensive composite score that reflects the overall suitability for irrigation. In this particular methodological framework, the total of all weights is designed to equal 100%, which allows for the effective multiplication of each raster’s weight by its respective scale, with the sums of these products subsequently aggregated across all layers to yield a robust suitability index.

The composite raster was then divided into four irrigation management suitability zones (High, Moderate, Low, and Very Low) to help guide site-specific irrigation management decisions. Similarly, GIS-based models have used a weighted overlay to construct irrigation water quality zoning maps^93^ and to assess groundwater irrigation quality in similar research sites^94^. The flexibility of this approach is its strength: weighting represents expert judgment or empirical sensitivity (by cross-validation or correlation analysis), and reclassification provides uniformity across rasters with varied units and ranges. Thus, the method enables the transparent and reproducible integration of different water-quality metrics into a single geographic framework to inform irrigation decisions in arid and semi-arid regions.

### Gibbs diagram

In order to investigate the natural processes that govern groundwater chemistry within the study area, the Gibbs diagram methodology established by Gibbs^95^ was utilized to differentiate among three primary processes: precipitation (rainfall) input, water-rock interaction, and evaporation concentration^96^. For each groundwater sample, two ionic ratios were calculated:-

2 $$\:Gibbs\:ratio\:\left(1\right)=\frac{{Na}^{+}}{{Na}^{+}+{Ca}^{2+}}$$

3 $$\:Gibbs\:ratio\:\left(2\right)=\frac{{Cl}^{-}}{{Cl}^{-}+{HCO}_{3}^{-}}$$

This methodology enabled the identification of whether evaporative concentration (Evaporation Dominance), interaction with aquifer minerals (Rock Dominance), or rainfall recharge (Precipitation Dominance) serves as the predominant factor affecting groundwater chemistry. This elucidates the temporal and spatial variations in groundwater quality and highlights areas that may be more vulnerable to the impacts of water-rock dissolution or increased salinity.

### Piper diagram

To determine the hydrochemical characteristics, a Piper diagram was created utilizing a pre-existing template in Microsoft Excel, specifically the Piper Diagram 2022-02.xltx^97^. This template systematically organizes ionic data, transforms them into equivalent percentages, and visually represents the outcomes in both the triangular and central fields, thereby facilitating the interpretation of geochemical patterns.

### IWQI calculation

**Fig. 2:**
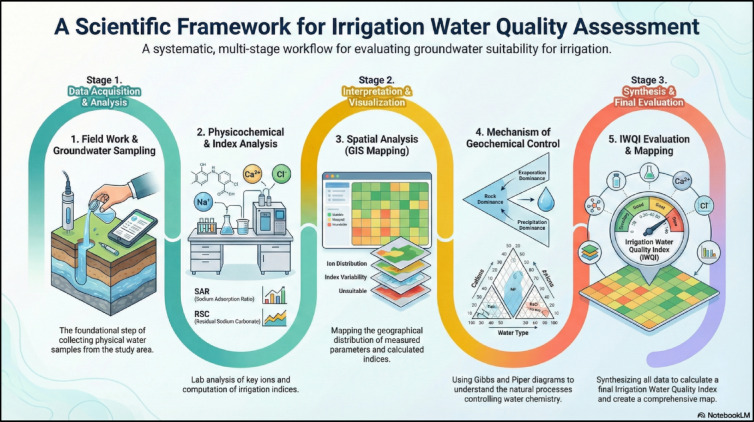
Methodological framework for irrigation water quality assessment (The image was generated using Google NotebookLM Infographic based on manuscript input sources https://notebooklm.google.com/notebook/6a2bb96e-7026-40c3-a46f-7c11913e161b).

In assessing the impact of various hydrochemical parameters on the quality of irrigation water and its suitability for agricultural purposes, the Irrigation Water Quality Index (IWQI) serves as an essential tool^77,98–100^. The methodological framework for irrigation water quality assessment is shown in (Fig. 2). Based on its computed values, the IWQI is typically categorized into five distinct groups:

1. Water classified as excellent (85–100) is considered to be of superior quality and is entirely suitable for irrigation without restrictions. It ensures that there will be no negative impacts on soil or crop productivity^101,102^.
2. Very good or slight restriction (IWQI = 70–85): This classification indicates water that is generally suitable for agricultural use. As noted by Şimşir, Yıldız^103^, it has only minor limitations that can be managed without significant agricultural inputs.
3. Good or moderate limitation (IWQI = 55–70): Water within this range may exhibit moderate limitations. This necessitates careful management practices to mitigate any potential adverse effects on crop yield and soil integrity^102^. This classification aids in making informed decisions regarding the use of irrigation water.
4. Permissible or high restriction (IWQI = 40–55): This quality category signifies water with considerable irrigation limitations. It typically requires remedial measures or effective management strategies to alleviate negative impacts^101^.
5. Inappropriate or severe restriction (IWQI = 0–40): Water in this category is regarded as highly unsuitable due to the risks associated with toxicity or salinity^103^.

In order to achieve a thorough evaluation and reliability of the index, the Irrigation Water Quality.

Index (IWQI) is generally developed through a four-step process^53,99,104,105^:

- Initially, the key factors that affect variations in irrigation water quality are identified utilizing principal component analysis and factor analysis (PCA/FA). As noted by^106–108^, only those components with eigenvalues exceeding one are preserved.
- Next, these factors help in determining the significant variables, thereby condensing the extensive dataset into a more digestible format that improves interpretation and applicability^109,110^.
- Subsequently, the Kaiser-Meyer-Olkin (KMO) statistic, which must exceed 0.5, along with Bartlett’s test of sphericity, were employed to confirm that the data were suitable for multivariate analysis in the context of assessing irrigation water quality. A statistically significant result from Bartlett’s test (*p* < 0.001) indicated that the dataset met these criteria. This supports the use of factor analysis in the formulation of the IWQI^111^.
- Finally, the last step involves modifying the initial weights (w_i_) until they sum to one, as per Eq. (4). This ensures that each variable contributes proportionately to the final index calculation^112,113^.

4 $$\:{W}_{i}={F}_{i}\times\:\frac{\sum\:_{j=1}^{k}{F}_{j}{A}_{ij\:}}{\sum\:_{j=1}^{k}\sum\:_{i=1}^{n}{F}_{j}{A}_{ij}}$$

Where Wi denotes the weight of the parameter, F signifies a component 1 auto value; i represents the count of physical and chemical parameters selected by the model, which ranges from 1 to n; j indicates the number of factors selected in the model, extending from 1 to k; and A_ij_ reflects the explanatory capacity of parameter i by factor j. The value of the water quality measurement parameter (qi) was subsequently established by utilizing Eq. (5), which is based on the water quality results determined in the laboratory and the tolerance limits specified in Table 3. The weight Wi of each parameter, as detailed in Table 4, is in accordance with the findings of Pivić, Maksimović^114^.

5 $$\:{{q}_{i}=\mathrm{q}}_{\mathrm{m}\mathrm{a}\mathrm{x}}-\left(\frac{\left({X}_{ij}-{X}_{inf}\right)*{q}_{iamp}}{{X}_{amp}}\right)$$

**Table 3:** Limiting values for parameters in the calculation of quality measurement (q_i_).

q_i_	EC (dS cm^− 1^)	SAR° (mmol^c^ L^− 1^)^1/2^	Na^+^	Cl^−^ (mmol^c^ L^− 1^)	HCO_3_
85–100	0.20 ≤ EC < 0.75	2 ≤ SAR° < 3	2 ≤ Na < 3	1 ≤ Cl < 4	1 ≤ HCO_3_ < 1.5
60–85	0.75 ≤ EC < 1.50	3 ≤ SAR° < 6	3 ≤ Na < 6	4 ≤ Cl < 7	1.5 ≤ HCO_3_ < 4.5
35–60	1.50 ≤ EC < 3.00	6 ≤ SAR° < 12	6 ≤ Na < 9	7 ≤ Cl < 10	4.5 ≤ HCO_3_ < 8.5
0–35	EC < 0.20 or EC ≥ 3.00	SAR° < 2 or SAR° ≥ 12	Na < 2 or Na ≥ 9	Cl < 1 or Cl ≥ 10	HCO_3_ < 1 or HCO_3_ ≥ 8.5

**Table 4:** The weights of the IWQI parameters.

Parameters	W_i_
EC	0.236
Na⁺	0.233
Cl⁻	0.230
HCO₃⁻	0.084
SAR°	0.217
Total	1.000

In accordance with Eq. (5), q_imax_ denotes the maximum value of qi within the class, xij represents the observed value of the parameter, and x_inf_ corresponds to the value that aligns with the lower limit of the class; Class amplitude (q_iamp_) and class amplitude (x_amp_) refer to the respective classes of the parameters. Finally, Eq. (6) was used to calculate the IWQI.

 

6 $$IWQI = \sum {_{{i = 1}}^{n} } q_{i} w_{i}$$

To enhance confidence in the spatial IWQI outputs, a brief sensitivity evaluation of the weighting scheme was considered. The assigned weights reflect the relative importance of salinity, sodicity, and specific ion toxicity based on established irrigation water quality guidelines. Minor perturbations in individual parameter weights did not lead to substantial changes in IWQI class distribution or spatial patterns, indicating that the model is robust and not overly sensitive to any single parameter. This stability confirms the reliability of the adopted weighting scheme and supports the consistency of the GIS-based irrigation suitability maps.

### Statistical analysis

In the present investigation, fundamental statistical methodologies were employed to explore and quantify the variability within the dataset, specifically focusing on metrics such as standard deviation (SD), measures of central tendency, including the mean and median, as well as the identification of the minimum and maximum values within the sample water.

The dataset comprising 41 wells was divided into two subsets: 25% was allocated for testing, while 75% was utilized for training. The models’ efficacy was corroborated by comparing the predicted and observed values. The performance of the employed model was assessed through metrics such as the root mean squared error (RMSE), additionally, the coefficient of determination (R^2^) were utilized.

7 $$\:RMSE=\sqrt{\frac{\sum\:_{i=1}^{n}{({P}_{i}-{O}_{i})}^{2}}{n}}$$

8 $$\:{R}^{2}\:\:\:\:=\:{\left[\frac{\sum\:_{i=1}^{n}\left({O}_{i}-\:\stackrel{-}{O}\right)\:\left({P}_{i}-\:\stackrel{-}{p}\right)\:\:}{\sqrt{\left(\sum\:_{i=1}^{n}{({O}_{i}-\:{\stackrel{-}{O}}_{i})}^{2}\right)}{\left(\sum\:_{i=1}^{n}({P}_{i}-\stackrel{-}{P})\right)}^{2})}\right]}^{2}$$

Where $\:{P}_{i}$ is the predicted value, $\:{O}_{i}$ is the observed value, and $\:n$ is the number of observations. For the purposes of conducting a comprehensive exploratory data analysis, the trial version of the Microsoft add-in program known as XLSTAT, which has been developed by the software company Addinsoft, was effectively utilized to facilitate the examination of the dataset. Furthermore, Principal Component Analysis (PCA) was systematically applied to the normalized data in order to uncover underlying connections and discernible differences that exist among the various variables included in the analysis.

By utilizing PCA, the complexity and dimensionality of a dataset comprising independent variables can be significantly reduced, thereby simplifying the data structure while retaining essential information. In the context of PCA, a collection of correlated variables is transformed through weighted linear combinations of the original variables into a new set of variables that are both orthogonal and.

uncorrelated, which enables a clearer interpretation of the data. These newly derived variables are referred to as Principal Components (PCs), as indicated in the research conducted by Dutta, Dwivedi^115^. To further enhance the analysis, hierarchical cluster analysis was performed, employing Euclidean distance as the principal similarity function to facilitate the completion of the clustering process, as demonstrated by the work of^116^.

## Results and discussions

Water in Egypt is a vital, existential issue intrinsically linked to national security, economic survival, and social stability, with policies shifting from supply-side expansion to demand management. Facing severe scarcity, Egypt’s management focuses on maximizing resources, enhancing efficiency in the agricultural sector (85% of usage), developing alternatives like desalination, and ensuring sustainable water security by 2050. The National Water Resources Plan aims to tackle scarcity through four pillars: rationalizing usage, improving water quality, developing new resources, and fostering supporting policies.

### Quality control

Assessing the ionic charge balance (E) equation represents the initial phase in the quality control process of the laboratory data. All percentage values are below 10%, as indicated by the Ion Charge Balance (E), which reflects the percentage discrepancy between the total cations and the aggregate of anions. Since it is under ± 10%, this indicates that the water analysis was precise and that the findings were deemed acceptable.

### Hydrogeochemical properties of irrigation water and the spatial distribution of quality parameters and indices

Groundwater quality fundamentally affects the possibility of agricultural activity and sustainable development in new reclamation areas, the most important of which in Egypt are all the desert lands adjacent to the Delta. These projects began in the late 1960 s and early 1970 s and continue to this day. With the continued agricultural expansion in these areas and the consumption of large quantities of groundwater from the aquifers in these areas, coupled with the lack of continuous government data related to groundwater levels and quality, as these studies are conducted over long periods of time, making it difficult to track changes in water quality and levels, this study focused on conducting a case study of groundwater quality in the western desert hinterland adjacent to the Delta, where most of the reclamation projects are concentrated and where the state will implement the New Delta project. The study clarified the impact of water quality on the selection of suitable crops, as most crops are suitable with salinity levels of less than 1000 ppm. However, with the excessive extraction of groundwater, this has led to an increase in salinity levels and reduced the opportunities for cultivating salt-sensitive crops or adopting a set of strategies to reduce the impact of salinity on plants. Therefore, this study provides important information for businessmen who want to invest in agricultural activity by identifying which areas are most suitable according to the type and quality of groundwater.

A total of 41 water samples representing the study area were collected, as detailed in Table 5. The analysis results of these water samples have been clarified, and the statistical information regarding the groundwater samples is summarized in Table 5. This table includes the pH, EC, TDS, and primary ions (Ca²⁺, Mg²⁺, Na⁺, K⁺, HCO₃⁻, Cl⁻, and SO₄²⁻), along with the minimum, maximum, mean, and standard deviation values. Additionally, calculated indices such as SAR, MAR, Na%, PI, RSC, and KR are also provided. The findings were compared with the irrigation water guidelines established by the Food and Agriculture Organization (FAO)^49,71^.

**Table 5:** Chemical, physical analysis, and summary statistics of groundwater samples in the study area.

Well No.	pH	EC (ds m^− 1^)	TDS (mg L^− 1^)	Na⁺	K⁺	Ca²⁺	Mg²⁺	HCO₃⁻	SO₄²⁻	Cl⁻	SAR	MR (%)	Na (%)	KR	PI (%)	RSC
				(Meq L^− 1^)												
1	7.07	1.03	659.00	5.10	0.85	2.50	1.35	0.10	3.20	7.00	3.68	35.06	60.71	1.32	60.52	−1.05
2	7.15	2.87	1836.00	14.70	2.00	8.00	4.00	0.30	8.40	20.00	6.00	33.33	58.19	1.23	57.11	−3.70
3	7.07	2.44	1561.00	13.90	1.50	7.00	3.00	0.30	6.40	18.00	6.22	30.00	60.63	1.39	60.45	−3.70
4	7.19	1.75	1120.00	9.80	1.20	5.00	2.50	0.20	4.30	13.00	5.06	33.33	59.46	1.31	59.23	−2.30
5	7.10	1.56	998.00	8.50	1.10	4.00	2.00	0.10	3.50	12.00	4.91	33.33	61.54	1.42	60.80	−1.90
6	7.08	2.90	1856.00	14.50	2.00	8.50	4.00	0.30	8.70	20.00	5.80	32.00	56.90	1.16	55.73	−4.20
7	7.02	0.73	467.00	4.30	0.50	1.50	1.00	0.10	1.20	6.00	3.85	40.00	65.75	1.72	67.89	−0.40
8	7.01	1.81	1160.00	11.00	1.00	4.10	2.00	0.20	3.90	14.00	6.30	32.79	66.30	1.80	66.94	−1.90
9	7.12	1.51	966.00	9.40	0.70	3.50	1.50	0.10	4.00	11.00	5.95	30.00	66.89	1.88	67.47	−1.90
10	7.18	1.55	990.00	9.50	0.80	3.60	1.60	0.10	4.20	11.20	5.89	30.77	66.45	1.83	66.78	−1.90
11	7.17	3.89	2490.00	17.90	4.00	11.50	5.50	0.30	13.60	25.00	6.14	32.35	56.30	1.05	52.86	−5.70
12	7.08	2.13	1360.00	9.30	2.00	6.50	3.50	0.20	6.10	15.00	4.16	35.00	53.05	0.93	50.50	−2.80
13	7.21	0.46	295.00	3.20	0.20	0.80	0.40	0.10	1.00	3.50	4.13	33.33	73.91	2.67	79.91	−0.30
14	7.10	1.66	1060.00	10.00	1.10	3.70	1.80	0.10	5.50	11.00	6.03	32.73	66.87	1.82	66.56	−1.80
15	7.23	2.45	1570.00	12.50	1.50	7.00	3.50	0.20	6.30	18.00	5.46	33.33	57.14	1.19	56.29	−3.30
16	7.15	2.50	1600.00	14.00	2.00	6.00	3.00	0.10	4.90	20.00	6.60	33.33	64.00	1.56	62.24	−2.90
17	7.22	3.77	2410.00	22.60	3.00	8.00	4.00	0.20	12.40	25.00	9.23	33.33	68.09	1.88	66.61	−3.80
18	7.10	0.80	514.00	4.00	1.00	2.00	1.00	0.10	1.90	6.00	3.27	33.33	62.50	1.33	61.66	−0.90
19	7.11	6.02	3850.00	35.00	3.00	15.00	7.00	0.30	19.70	40.00	10.55	31.82	63.33	1.59	62.36	−7.70
20	7.02	0.33	211.00	2.00	0.20	0.80	0.30	0.10	0.70	2.50	2.70	27.27	66.67	1.82	74.72	−0.40
21	7.15	0.68	433.00	3.80	0.50	1.50	1.00	0.10	2.20	4.50	3.40	40.00	63.24	1.52	65.34	−0.40
22	7.19	0.63	404.00	3.60	0.40	1.40	0.90	0.10	2.00	4.30	3.36	39.13	63.49	1.57	66.38	−0.40
23	7.15	1.23	790.00	5.30	1.00	3.50	2.50	0.10	3.70	8.50	3.06	41.67	51.22	0.88	49.70	−0.90
24	7.22	1.18	760.00	5.20	0.70	3.20	2.10	0.10	3.60	8.20	3.19	39.62	52.68	0.98	52.54	−1.00
25	7.64	7.01	4486.00	49.73	0.36	18.14	17.98	4.22	3.03	51.60	11.70	49.78	58.10	1.38	60.32	4.06
26	7.90	0.95	608.00	6.55	0.15	0.72	1.10	2.20	1.09	5.23	6.87	60.44	78.64	3.60	95.98	2.58
27	7.05	4.71	3014.00	28.00	4.10	10.00	5.00	0.30	8.80	38.00	10.22	33.33	68.15	1.87	66.39	−4.70
28	7.12	4.27	2732.00	26.00	3.70	9.00	4.00	0.30	8.70	33.70	10.20	30.77	69.56	2.00	68.07	−4.70
29	7.15	4.10	2624.00	25.40	3.60	8.50	3.50	0.20	7.80	33.00	10.37	29.17	70.73	2.12	69.11	−4.80
30	7.15	4.44	2842.00	24.00	3.40	11.00	6.00	0.40	14.00	30.00	8.23	35.29	61.71	1.41	60.08	−4.60
31	7.22	4.75	3040.00	25.50	3.50	12.00	6.50	0.50	15.00	32.00	8.38	35.14	61.05	1.38	59.56	−5.00
32	7.17	1.07	683.00	4.70	1.00	3.00	2.00	0.10	3.60	7.00	2.97	40.00	53.27	0.94	51.71	−0.90
33	7.12	1.08	688.00	4.60	1.10	3.10	2.10	0.10	3.70	7.10	2.85	40.38	52.29	0.88	50.17	−0.90
34	7.10	1.05	669.00	4.50	1.00	2.90	1.90	0.10	3.30	7.00	2.90	39.58	53.40	0.94	51.79	−0.90
35	7.19	0.78	499.20	3.99	0.11	2.40	1.70	1.11	1.98	2.65	2.79	41.46	50.00	0.97	62.34	0.41
36	7.32	3.56	2278.40	17.60	0.89	9.70	6.70	5.20	7.90	17.60	6.15	40.85	53.00	1.07	58.47	2.20
37	7.31	3.99	2553.60	16.81	1.32	10.90	6.32	5.80	9.80	21.90	5.73	36.70	51.29	0.98	56.47	1.22
38	7.44	2.91	1862.40	11.80	0.77	7.90	5.10	5.70	3.50	9.50	4.63	39.23	49.16	0.91	57.21	2.90
39	7.10	1.90	720.00	4.60	0.90	3.00	2.00	0.10	3.50	6.80	2.91	40.00	52.38	0.92	51.21	−0.90
40	7.25	2.25	1440.00	12.20	0.65	4.90	3.70	3.20	6.90	13.10	5.88	43.02	59.91	1.42	67.25	2.00
41	7.21	1.23	787.20	5.29	0.43	3.10	2.80	2.32	2.80	3.98	3.08	47.46	49.23	0.90	60.89	2.02
Minimum	7.01	0.33	211.00	2.00	0.11	0.72	0.30	0.10	0.70	2.50	2.70	27.27	49.16	0.88	49.70	−7.70
Maximum	7.90	7.01	4486	49.73	4.10	18.14	17.98	5.80	19.70	51.60	11.70	60.44	78.64	3.60	95.98	4.06
Average	7.46	3.67	2348.50	25.87	2.11	9.43	9.14	2.95	10.20	27.05	7.20	43.86	63.90	2.24	72.84	−1.82
Std. deviation	7.18	2.34	1485.04	12.69	1.44	5.83	3.36	0.87	5.78	15.70	5.63	36.57	60.42	1.45	61.89	−1.59
FAO standard range for irrigation water	-	< 0–3	< 2000	< 40	< 2	< 20	< 5	< 10	< 20	< 30	-	-	-	< 1	-	-

**Fig. 3:**
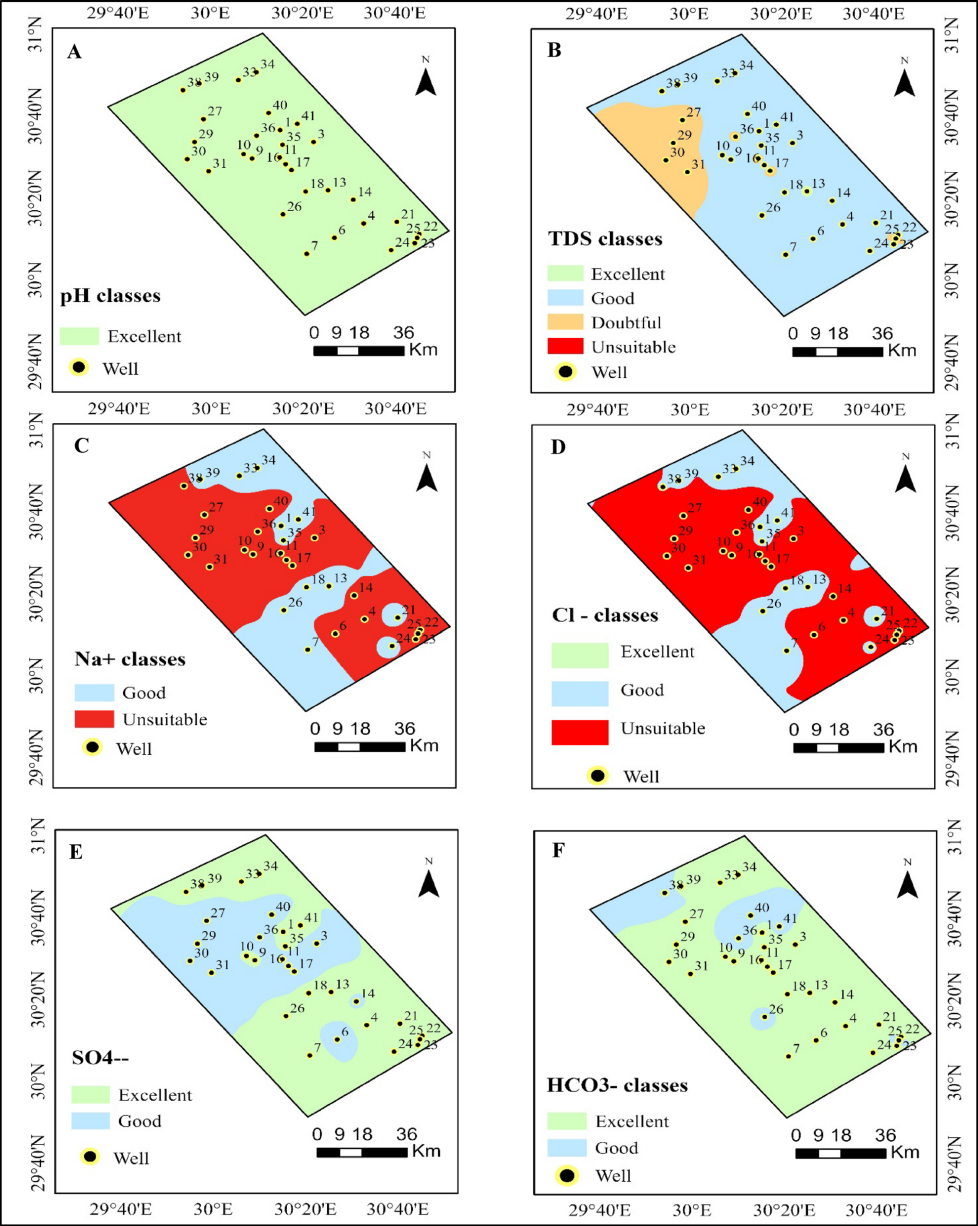
Spatial distribution and classification of groundwater quality parameters for irrigation in the study area.

Based on FAO standard range for irrigation water criteria, the entire study area (7486.83 km^2^) is classified as excellent, as the pH of the groundwater ranges from 7.01 to 7.90 (mean 7.18). Most crops can be irrigated since the water in this region is neutral to slightly alkaline, as shown in Table 6 and (Fig. 3A). This wide range of high-quality pH values indicates that neither alkalinity nor acidity poses a limiting factor. However, as illustrated by the regional map, slightly alkaline water resulting from carbonate dissolution and agricultural inputs may increase the risk of sodicity if it is combined with elevated Na⁺ levels^77,117,118^. The average TDS value in groundwater is 1,485 mg L^− 1^, with a range spanning from 211 to 4,486 mg L^− 1^. The high TDS values could be attributed to sea water intrusion due to high abstractions^119^.

**Table 6:** Classification of irrigation water quality parameters with their corresponding spatial coverage in study area.

Parameters	Class	Area (Km^2^)	Area (%)
pH	Excellent	7486.83	100.00
TDS	Excellent	17.63	0.25
	Good	5938.44	83.21
	Doubtful	1170.46	16.40
	Unsuitable	10.17	0.14
Cl^−^	Excellent	7.09	0.10
	Good	2316.61	30.94
	Unsuitable	5163.09	68.96
Na^+^	Good	2810.79	37.54
	Unsuitable	4675.99	62.46
SO₄²⁻	Excellent	4363.31	58.28
	Good	3123.47	41.72
HCO₃⁻	Excellent	6330.13	84.55
	Good	1156.65	15.45

Based on the spatial classification (Fig. 3B) and Table 6, 0.25% of the area (17.63 km²) falls into the excellent category, 83.21% is classified as good (5938.44 km²), 16.40% is categorized as doubtful (1170.46 km²), and merely 0.14% is deemed Unsuitable (10.17 km²) (Table 6). However, concentrations nearing the upper limit may pose challenges for crops that are sensitive to salinity^120^. The EC value varied from 0.33 to 7.01 dS m^− 1^, with an average of 2.34 dS m^− 1^. According to FAO criteria, approximately 50% of the samples are regarded as marginally suitable for irrigation (0–3 dS m^− 1^). Consistent with findings from previous studies in Egyptian aquifers^77^, samples that exceed 3 dS/m are subject to significant salinity restrictions^104^. It is advisable to cultivate crops such as barley, cotton, and beets that can tolerate salt if the EC exceeds 3 dS m^− 1^.

Avoid planting sensitive crops like leafy vegetables and legumes. Concerning the primary cations, sodium ion (Na^+^) concentrations range from 2.00 to 49.73 meq L^− 1^, with an average of 12.69 meq L^− 1^. According to the spatial map (Fig. 3C), 37.54% of the area (2810.79 km^2^) falls into the good category, while the majority, 62.46% (4675.99 km^2^), is classified as unsuitable for irrigation due to the risk of sodicity (Table 6). Elevated levels of Na^+^ pose a significant threat to soil permeability and structure, especially in areas rich in clay. This observation aligns with recent research conducted in the Nile Delta, which identified sodicity as the primary obstacle to irrigation suitability^102^.

The mean concentration of calcium ions (Ca^2+^) is 5.83 meq L^− 1^, with values ranging from 0.72 to 18.14 meq L^− 1^. Magnesium ion (Mg²⁺) concentrations average 3.36 meq L^− 1^, with a range of 0.30 to 17.98 meq L^− 1^. Both Ca^2+^ and Mg²⁺ typically remain within acceptable limits, with thresholds of fewer than 20 and 5 meq L^− 1^, respectively. However, certain concentrations of Mg²⁺ exceed the recommendations set by the FAO. Prolonged irrigation can adversely affect soil structure, reduce permeability, and enhance colloid dispersion^121^. Potassium (K⁺) generally poses less of a structural risk within this concentration range, as plants can readily absorb it, thereby limiting its accumulation in soil solutions^122^. The potassium concentrations measured ranged from 0.11 to 4.10 meq L^− 1^, with a mean of 1.44 meq L⁻¹. Nevertheless, many irrigation water guidelines suggest that K⁺ should be carefully monitored when it exceeds approximately 2.0 meq L^− 1^ due to the potential for nutrient imbalance or interference with other cations^49^. While the majority of the wells in our dataset remained below that threshold, a few exceeded it.

These results could be attributed to local geological sources, fertilizer leaching, or irrigation return flows, which may adversely affect soil permeability and osmotic conditions in root zones^121^. Similar observations have been reported in semi-arid aquifers in North Africa and South Asia, where a decline in water suitability for irrigation has been associated with elevated K⁺ concentrations in certain wells^77,123^.

Consequently, although potassium does not dominate the groundwater hazard profiles in our study, the health of the soil and the performance of crops can be safeguarded by conducting regular monitoring of wells with K⁺ levels exceeding 2.0 meq L⁻¹ and by employing mitigation strategies such as crop selection, blending, and balanced nutrient management. When compared to previous research, the findings of this study align with the general trends observed in the Nile Delta. Most samples exhibit Ca^2+^ and Mg^2+^ values that remain within safe limits for irrigation. Therefore, their immediate negative impacts on soil quality are mitigated. Conversely, Na^+^ emerges as the most critical element, as it often exceeds the recommended thresholds. This situation jeopardizes the sodicity and structure of the soil. In contrast, K⁺ serves as a secondary indicator primarily linked to regional agricultural practices and does not significantly influence the quality of irrigation water when compared to sodium.

For the anions, chloride levels range from 2.50 to 51.60 meq L^− 1^, with an average of 15.70 meq L^− 1^. The distribution map (Fig. 3D) indicates that 30.94% of the area (2316.61 km²) falls within the good category, 0.10% of the area (7.09 km²) is classified as excellent, while the majority, 68.96% (5163.09 km²), is deemed unsuitable due to elevated chloride concentrations (Table 6). The observed chloride enrichment is likely attributed to the combined influence of over-pumping–induced vertical mixing with deeper saline groundwater and leaching of salt-rich lagoonal and marine clay layer^64^. This suggests that chloride is a significant limiting factor for irrigation water in the study region. Chloride-sensitive crops may suffer when chloride levels exceed 30 meq L^− 1^ in certain samples, especially under dry or semi-dry conditions where salt leaching is restricted^103^. These findings align with recent studies conducted in North Africa and the Nile Delta, which also revealed that sensitive crops such as citrus and legumes exhibit reduced productivity in the presence of chloride accumulation^124,125^. As noted by Elsharkawy^126,127^, this reduces the likelihood of complications related to carbonate or a high residual sodium index (RSC).

The SO₄²⁻ distribution shows that groundwater quality ranges between Excellent and Good, with no areas classified as Unsuitable. Elevated sulfate concentrations are spatially limited and likely linked to the dissolution of gypsum or anhydrite minerals within the aquifer matrix. Overall, sulfate levels remain within acceptable limits, indicating minimal health or agricultural concerns. The spatial pattern suggests natural geochemical control rather than anthropogenic pollution, supporting the dominance of lithological influence on groundwater chemistry (Fig. 3E). The sulfate concentrations range from 0.70 to 19.70 meq L^− 1^, with a mean of 5.78 meq L^− 1^. According to Tables 6, 41.72% (3123.47 km²) of the area is classified as good, while 58.28% (4363.31 km²) falls into the excellent category, as shown in the maps (Fig. 3E). All values remain within FAO guidelines, indicating minimal risk from sulfates in irrigation water.

The HCO₃⁻ classification map demonstrates that most groundwater samples fall within the excellent class, with minor zones classified as Good. High bicarbonate concentrations are typical of groundwater systems influenced by carbonate weathering and CO₂-rich recharge water (Fig. 3F). The dominance of bicarbonate confirms the role of carbonate rocks and soil CO₂ dissolution in shaping groundwater chemistry. Such conditions are generally beneficial, as bicarbonate contributes to buffering capacity and stabilizes pH within a favorable range. Bicarbonate levels range from 0.10 to 5.80 meq L^− 1^, with an average of 0.87 meq L^− 1^. Figure 3F illustrates that 84.55% (6330.13 km²) of the area is categorized as excellent, while the remaining 15.45% (1156.65 km²) is classified as good (Table 6).

The calculated indices have revealed potential limitations that warrant further examination and consideration. HCO_3_^–^ concentrations are generally low across the study area, with most samples falling within the excellent irrigation-quality category (Fig. 3F; Table 6). Slightly elevated levels are limited to localized zones, suggesting a minor influence of carbonate weathering and limited soil–water interaction under prevailing arid conditions.

**Fig. 4:**
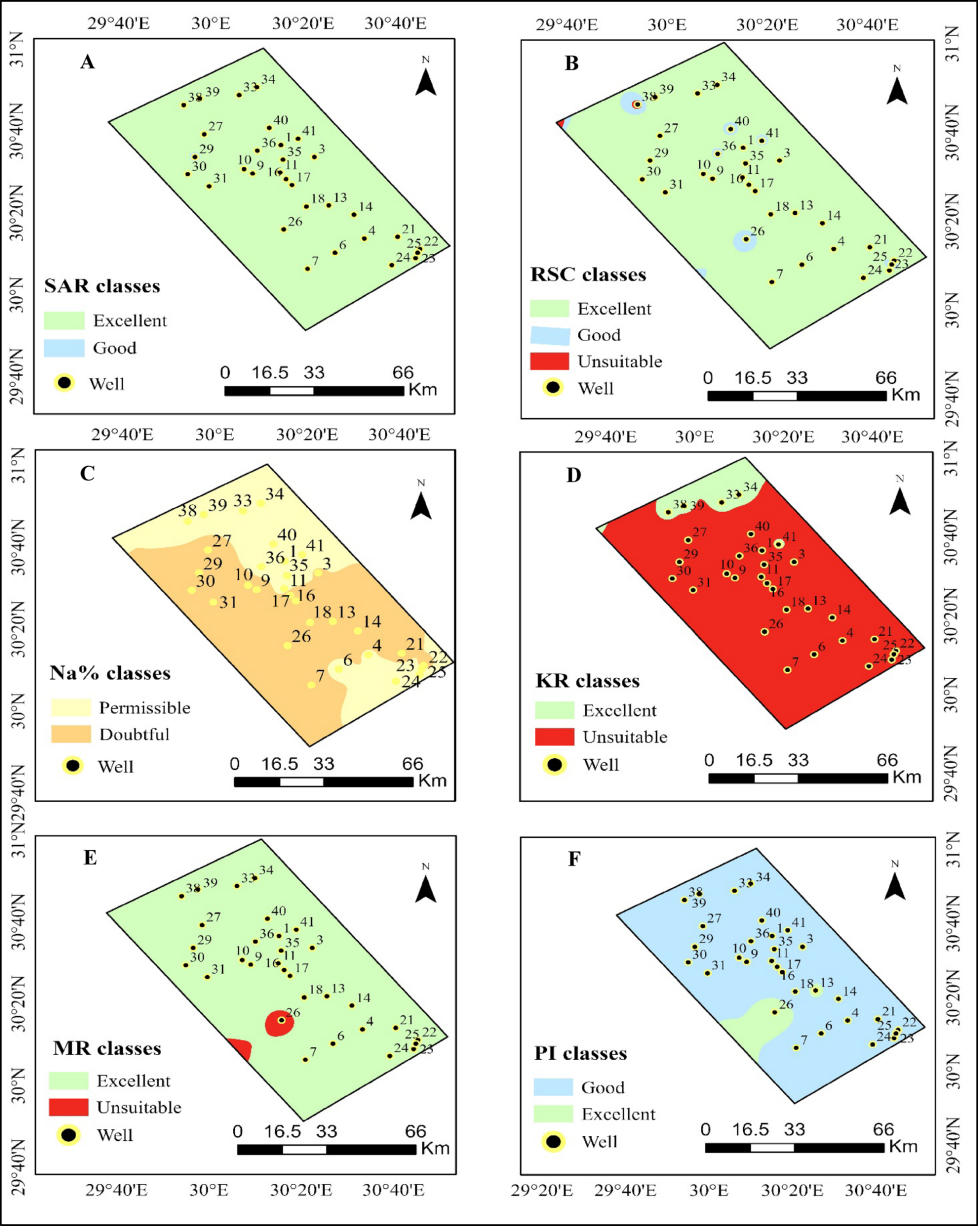
Spatial distribution and classification of groundwater quality indices for irrigation in the study area.

The results indicate that a significant majority of the groundwater samples could indeed be utilized effectively for irrigation purposes, as inferred from the computed Sodium Adsorption Ratio (SAR) values, which exhibited a range from 2.69 to 11.70 meq L^− 1^, with a calculated average of 5.62 meq L^− 1^. When these values were translated onto the spatial distribution map presented in (Fig. 4A), an overwhelming 99.71% of the area, which corresponds to an impressive 7465.04 km^2^, was classified as Excellent, while only a minuscule 0.29%, equating to 21.74 km^2^, was designated as Good, as demonstrated in Table 7. The overall sodium hazard within the study area is suggested to be quite low, primarily due to the predominance of water samples exhibiting low SAR values. Nevertheless, it is imperative to exercise caution, particularly in regions characterized by fine-textured clay soils, as prolonged irrigation practices utilizing water with elevated SAR levels may significantly impair soil permeability, as indicated by previous research conducted by Abbasnia, Radfard^101,128^.

**Table 7:** Classification of irrigation water quality indices with their corresponding spatial coverage in study area.

Indices	Class	Area(Km^2^)	Area%
SAR	Excellent	7465.04	99.71
	Good	21.74	0.29
RSC	Excellent	7273.84	97.16
	Good	192.14	2.57
	Unsuitable	20.80	0.28
Na%	Permissible	2937.71	39.24
	Doubtful	4549.07	60.76
KR	Excellent	644.97	8.62
	Unsuitable	6841.81	91.39
MR	Excellent	7340.25	98.04
	Unsuitable	146.53	1.96
PI	Excellent	516.64	6.90
	Good	6970.14	93.10

Similar observations were documented in China, where the majority of SAR values fell into the categories of “excellent” or “good,” thereby illustrating that irrigation water was generally deemed suitable for agricultural applications, with only a handful of exceptions noted, as reported by Xie, Ning^46^. The values associated with the RSC, or Residual Sodium Carbonate, predominantly exhibited negative attributes, demonstrating a range that spanned from a minimum of −7.7 to a maximum of 4.06 meq L^− 1^, culminating in a mean value calculated at approximately − 1.59 meq L^− 1^, which collectively suggests that the environmental conditions present in the area are rather conducive and favorable concerning the potential hazards posed by carbonate interactions.

An examination of the spatial distribution map, which is illustrated in (Fig. 4B), reveals that an overwhelming majority, specifically 97.16% of the total area, equivalent to about 7273.84 square kilometers, has been classified under the Excellent category, while a smaller proportion, constituting 2.57%, which translates to approximately 192.14 square kilometers, has been designated as Good, and a mere 0.28%, or 20.80 square kilometers, falls into the Unsuitable category, as delineated in Table 7. This discernible pattern serves to reinforce the notion that the long-term sustainability of utilizing groundwater resources for irrigation purposes under the prevailing conditions is well supported, thereby indicating that the risks associated with carbonate-related soil degradation remain relatively low throughout the region, as corroborated by the findings put forth by Şimşir, Yıldız^103,129^.

The percentage of sodium (Na%) values demonstrated a variation ranging from 49.16% to 78.64%, culminating in a mean value of 60.41%. The spatial distribution map illustrated in (Fig. 4C) indicates that a significant 60.76% of the area, which translates to 4549.07 km^2^, is classified as doubtful, while the remaining 39.24%, amounting to 2937.71 km^2^, is categorized as permissible, as detailed in Table 7. This classification underscores the fact that sodium toxicity represents a considerable concern within a substantial portion of the research area. This issue is particularly pronounced in recently reclaimed lands characterized by clay-rich soils, where the prolonged irrigation with water containing high Na% levels may exacerbate soil sodicity and lead to structural degradation. Comparable findings have been documented in other semi-arid regions, where Na% levels were identified as a significant limiting factor affecting the suitability of irrigation practices, as evidenced by studies conducted by^77^, Ali, Ebrahem^130^.

In the concluding remarks, it is pertinent to note that the values for KR, which represents Sodium Absorption Ratio, were observed to range from as low as 0.88 to as high as 3.60 meq L^− 1^, with the computed mean resting at approximately 1.45 meq L^− 1^, which provides critical insights into the sodium concentration levels in the groundwater (Fig. 4D).

The spatial distribution map, as depicted in (Fig. 4D), illustrates that a staggering 91.39% of the area, corresponding to about 6841.81 square kilometers, falls into the Unsuitable classification, while a significantly smaller fraction, precisely 8.62% of the area, which measures around 644.97 square kilometers, is categorized as Excellent, as detailed in Table 7. This distribution suggests that sodium concentrations are predominantly higher than those of calcium and magnesium in the vast majority of the collected samples, which could potentially lead to adverse effects such as diminished soil permeability and increased dispersion issues, particularly in clay-rich soils that are typically encountered in areas that have been recently reclaimed for agricultural purposes.

Comparable studies conducted in arid and semi-arid regions have also indicated KR values that exceed critical thresholds, thereby underscoring the vital importance of effectively managing sodium concentrations in irrigation water, as evidenced by the research conducted by Thirumoorthy, Velusamy^131,132^. The findings presented by Soomro, Hao^132^, report KR values ranging from 0.2 to 4.1, which demonstrate that while some samples indeed surpassed the critical sodium value, the majority remained within acceptable parameters, thus aligning closely with the results obtained in our study. Consequently, the implications of the KR results within the studied area serve to highlight the pressing necessity of mitigating sodium-related risks in order to ensure the sustainable utilization of reclaimed agricultural lands. While it is true that the water samples generally conform to various quality indicators, the elevated percentages of sodium present emphasize the critical need for diligent salinity management practices, which may include strategic crop selection, the application of soil amendments, and meticulously organized irrigation schedules, all aimed at alleviating the potential risks associated with sodicity.

The Magnesium Ratio (MR) values exhibited a range from 27.27 to 60.44, with a computed mean of 36.57. An examination of the spatial distribution map showcased in (Fig. 4E) reveals that an impressive 98.04% of the study area, which encompasses 7340.25 square kilometers, is categorized as Excellent, whereas a mere 1.96%, corresponding to 146.53 square kilometers, falls into the Unsuitable category, as summarized in Table 7. This distribution suggests that magnesium poses a low to moderate risk within the region. However, it is essential to recognize that if irrigation practices persist over extended periods in localized areas where MR levels are elevated, there may be potential risks to the soil structure that must be addressed. Nevertheless, the overall distribution indicates that, in stark contrast to sodium, magnesium does not emerge as a critical limiting factor in the context of irrigation suitability.

The Permeability Index (PI) values ranged from 49.70% to 95.97%, with an average value of 61.89%. As evidenced by the spatial distribution map illustrated in (Fig. 4F), a small proportion of 6.90% of the area, which translates to 516.64 square kilometers, has been classified as Excellent, while a substantial 93.10%, amounting to 6970.14 square kilometers, has been designated as Good, as presented in Table 7.

Recent reports from the Nile Delta indicate that bicarbonate concentrations do not pose a limiting factor for irrigation suitability in the study area^133^. Monitoring chloride ions is essential in this context as it serves as the main indicator of water quality. Unlike previous studies, it has become clear that the significant variability observed in these samples correlates with elevated chloride levels at certain locations, whereas sulfate and bicarbonate concentrations remain within acceptable thresholds.

This finding indicates that, when sodium risks are meticulously managed, the irrigation water is generally considered appropriate for the maintenance of soil permeability. Similar observations have been reported for reclaimed lands in the Nile Delta, where careful management practices concerning crops and soil were deemed necessary despite the PI values indicating a good level of suitability, as documented by^1,77^, Ali, Ebrahem^130^.

### Mechanism of geochemical control

**Fig. 5:**
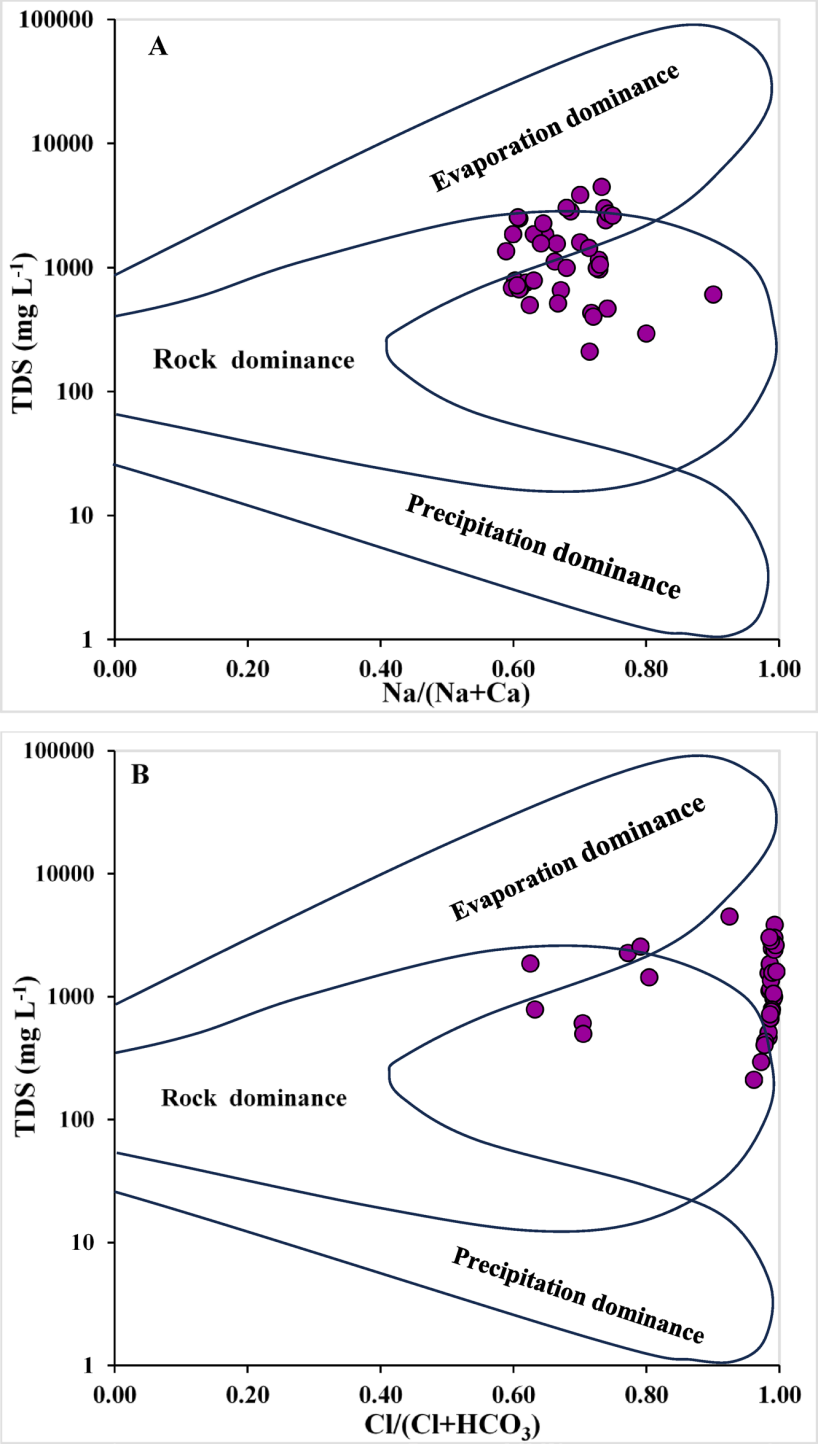
The Gibbs diagram illustrated the mechanism of geochemical control, (**A**) the relationship between total dissolved solids (TDS) and the ratios of Na^+^/(Na^+^ + Ca^2+^), and (**B**) the relationship between total dissolved solids (TDS) and the ratios of Cl^−^/(Cl^−^ + HCO₃⁻).

The Gibbs diagram serves as an invaluable method for elucidating the geochemical processes that govern the quality of groundwater resources. As articulated by Gibbs^95^, this diagram effectively illustrates the intricate relationship that exists between total dissolved solids (TDS) and the ratios of sodium ions to the sum of sodium and calcium ions (Na^+^/(Na^+^ + Ca^2+^)) (Fig. 5A) as well as the ratios of chloride ions to the sum of chloride and bicarbonate ions (Cl^−^/(Cl^−^ + HCO₃⁻)), as depicted in (Fig. 5B). It was observed that the majority of the water samples analyzed fell within the Rock Dominance range, while a subset of samples was identified within the Evaporation Dominance category. This observation suggests that the primary source of the dissolved ions can be attributed to the interactions occurring between the groundwater and the geological formations present, such as the melting of carbonates, silicates, and gypsum deposits, while the phenomenon of evaporation evidently contributes to heightened salinity levels in certain samples that exhibited elevated concentrations.

These findings align with those presented by Hussein, El Maghraby^1^, who concluded that evaporation processes, especially in semi-arid areas, exert a secondary influence on the groundwater of the Nile Delta, which is predominantly affected by the melting of carbonate and silicate rocks^134,135^. This interpretation is further supported by PHREEQC saturation index calculations, showing that most groundwater samples are under saturated with respect to carbonate minerals (calcite, aragonite, and dolomite) and sulfate minerals (gypsum and anhydrite), reflecting ongoing mineral dissolution along the groundwater flow paths. In contrast, chloride minerals (halite and sylvite) are consistently far from saturation, confirming that chloride enrichment is not controlled by evaporite precipitation but rather by conservative processes such as mixing with deeper saline groundwater and leaching from salt-rich lagoonal and marine clay deposits. The concurrence between Gibbs-based interpretation and SI results demonstrates that groundwater salinization is primarily driven by lithological controls and hydraulic connectivity, with evaporation playing a subordinate role under arid to semi-arid conditions and intensified groundwater abstraction.

### Piper diagram

**Fig. 6:**
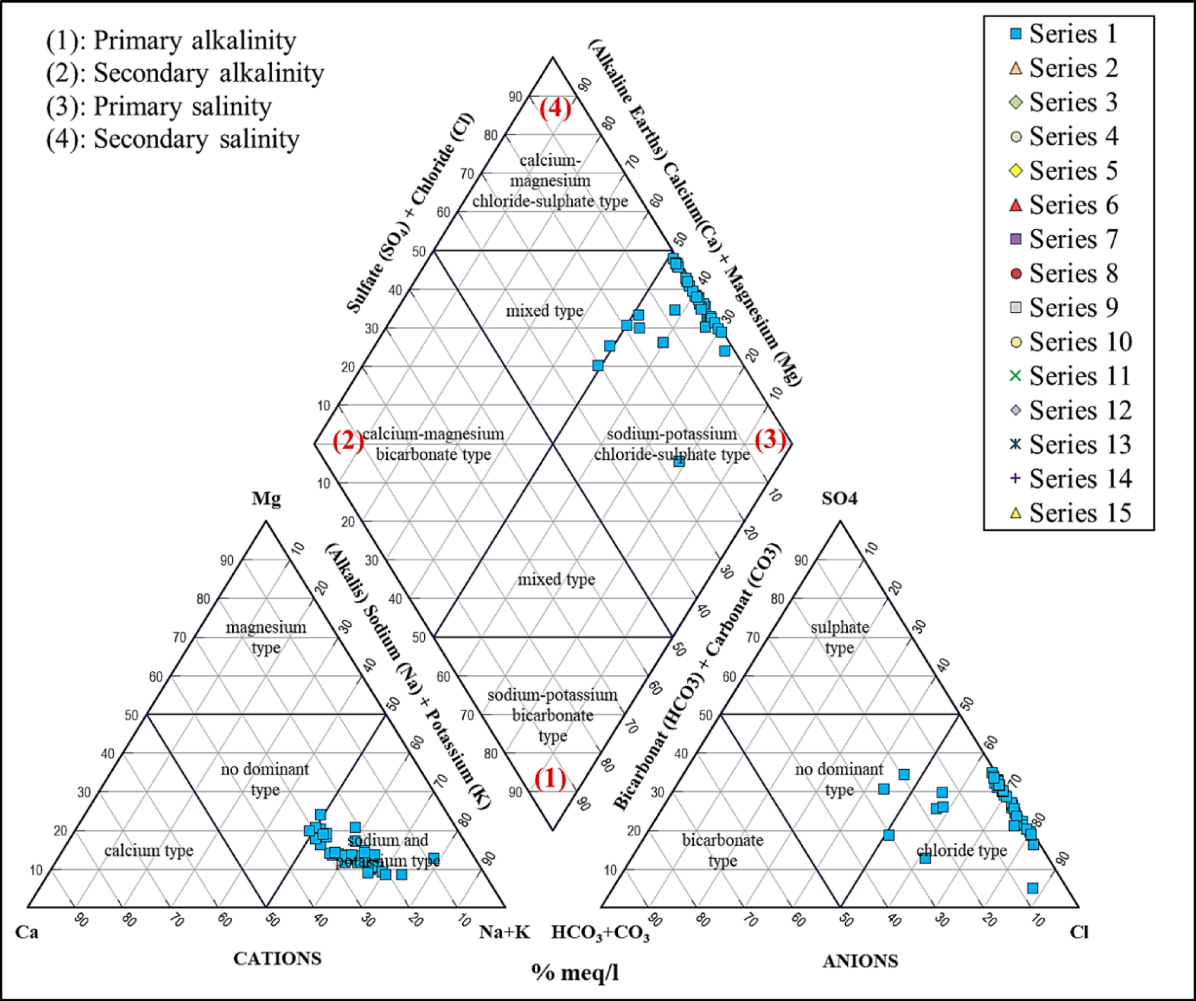
Piper diagram illustrating the hydrochemical faces of groundwater in the study area, highlighting the dominance of Ca–Mg–HCO₃ type, the presence of mixed faces, and limited occurrence of Na–Cl type.

In the Piper diagram illustrated that the sample distribution is clearly evident: Approximately 76% of the samples are located within the “Alkaline-Earth + Weak Acidic Anions” zone (Fig. 6), indicating the significant impact of carbonate and silicate rock measurements, along with the presence of recharge water that resembles river characteristics (showing clear compatibility with Nile water); About 22% of the samples are located in the mixed area (Mixed Faces), implying the convergence of diverse water sources or local anthropogenic influences, while a single sample (~ 2%) is exclusively identified within the initial salinity range, which is characterized by base alkalis and strong anions (Na–Cl type). Based on these findings, the primary source—the melting of minerals—predominates, with evaporation and human activities serving as secondary factors that modify the chemical composition of certain samples. These results carry important hydrochemical implications for the management of irrigation water and the soil’s vulnerability to sodicity.

This observed distribution corresponds with findings from studies conducted in the Nile Delta and other coastal areas, which have shown that the majority of groundwater wells display Ca - Mg - HCO₃ (arising from the dissolution of carbonate and silicate), with only a few groups transitioning towards Na - CL due to evaporative concentration or seawater intrusion^121,136^. Hydrochemical analyses, including Piper diagrams, identified two water types: Ca–Mg–Cl (50%) and Ca–Cl (50%). the groundwater’s mineralization is primarily attributed to water–rock interactions, such as the dissolution of silicate rocks, halite, and gypsum, along with cation exchange^137^.

### Cross-validation of interpolation

Cross-validation was used to examine the validity of geographical interpolation for various groundwater quality parameters and indices. Table 8 summarizes the results, including the regression equations (predicted versus observed), coefficient of determination (R^2^), and root mean squared error (RMSE) for each parameter/index obtained for all sample points.

**Table 8:** Cross-validation results of groundwater quality interpolation.

Parameters and indices	Equation	*R*^2^	RMSE
pH	y = 3.75 + 0.47 x	0.063	0.11
TDS	y = −38.0 + 0.9 x	0.9	295.46
Cl	y = 4.0 + 0.63 x	0.86	4.05
Na	y = 1.83 + 0.64 x	0.93	4.11
SO_4_	y = 3.07 + 0.41 x	0.47	2.93
HCO_3_	y = 2.6 + −2.1 x	0.073	2.44
SAR	y = 2.5 + 0.43 x	0.37	1.55
RSC	y = −2.75 + −0.59 x	0.43	3.13
Na%	y = 79.2 + −0.39 x	0.06	7.58
KR	y = 1.33 + −0.125 x	0.01	0.43
MR	y = 31.8 + 0.101 x	0.02	4.21
PI	y = 36.3 + 0.34 x	0.06	6.82

Total dissolved solids (TDS) exhibited one of the most elevated R^2^ values (0.90) alongside a corresponding RMSE of 295.46, signifying that the interpolation effectively captured the overall spatial variability of total dissolved solids. Chloride (Cl⁻) revealed a robust correlation (R^2^ = 0.86, RMSE = 4.05), implying that it sufficiently represents spatial distributions of chloride. The concentration of sodium (Na⁺) within the aquifer demonstrates a high degree of predictability, as indicated by R^2^ = 0.93 and RMSE = 4.11. Conversely, the other input variables, including pH, HCO₃⁻, Na%, PI, MR, and KR, exhibited R^2^ values below 0.1 with correspondingly high RMSE, suggesting that the establishment of predictable relationships was infeasible due to potential regional heterogeneity or noise interference. The sodium adsorption ratio (SAR) displayed moderate predictability metrics (R^2^ = 0.37, RMSE = 1.55), while the residual sodium carbonate (RSC) also indicated intermediate predictability with R^2^ = 0.43 and RMSE = 3.13, thereby suggesting that the reliability of interpolations remained at a moderate level.

The outcomes derived from the cross-validation indicate that conservative parameters (such as Na⁺, Cl⁻, TDS) exhibit effective interpolation due to smooth spatial gradients and minimal influence from local phenomena. In contrast, local biochemical processes significantly affect parameters such as HCO₃⁻, pH, PI, and Na%, resulting in substantial geographical variance and suboptimal interpolation performance. This observation is consistent with prior research indicating that indices influenced by local redox conditions, microbial activity, or surface inputs in near-surface environments pose challenges for accurate interpolation in groundwater quality mapping^138–140^. Recent regional studies have established that TDS and Na⁺ (or EC and Na⁺) are the most effectively interpolated variables for assessing groundwater in arid and semi-arid regions, whereas composite indices (such as IWQI, PI) or ratios that are sensitive to minor fluctuations are associated with high uncertainties^37,141,142^. From the cross-validation findings, we delineate two pivotal conclusions:.

1. Greater emphasis should be placed on TDS, Na⁺, and Cl⁻ layers in spatial forecasts during the creation of high-quality maps or zoning.
2. It is advisable to implement a sample or (spatial) local correction (e.g., Co-kriging, kriging of residuals, or incorporation of secondary variables) for the conclusive decision-making maps or management of parameters or indices with low predictability.

### Wilcox diagram

**Fig. 7:**
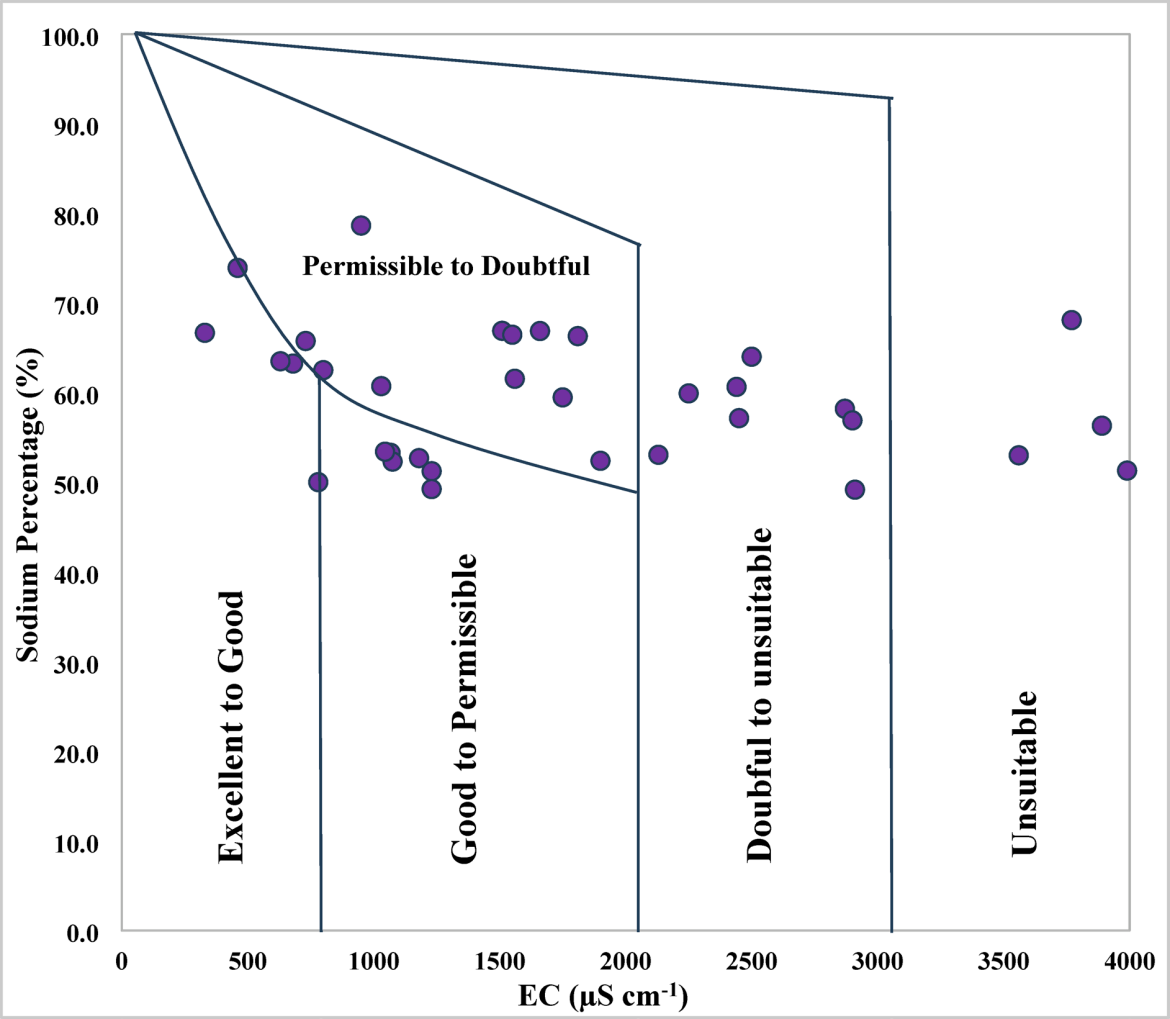
Evaluation of irrigation water quality using Wilcox diagram.

The Wilcox diagram serves as a conventional method for evaluating the quality of irrigation water by examining the correlation between sodium percentage (Na%) and electrical conductivity (EC) (Fig. 7). This classification instrument aids in identifying the levels of salinity and sodicity risks, thus offering valuable insights into their potential effects on soil properties and crop yield. Based on the Wilcox diagram in conjunction with Na%-based classification (Table 2), a modest 17.07% of water samples are categorized as excellent to good, while another 17.07% fall into the good to permissible category. A more significant portion, 31.71%, is classified as permissible to doubtful, and 9.76% are categorized as doubtful to unsuitable. Importantly, 24.39% of the samples are deemed unsuitable for irrigation.

As illustrated in (Fig. 7), it is evident that the majority of samples are situated within the permissible to doubtful range, indicating that while many wells may not be optimal, they can still be utilized with caution. Based on these findings, the primary source—the melting of minerals—predominates, with evaporation and human activities serving as secondary factors that modify the chemical composition of certain samples. These results carry important hydrochemical implications for the management of irrigation water and the soil’s vulnerability to sodicity.

This observed distribution corresponds with findings from studies conducted in the Nile Delta and other coastal areas, which have shown that the majority of groundwater wells display Ca - Mg - HCO₃ (arising from the dissolution of carbonate and silicate), with only a few groups transitioning towards Na - CL due to evaporative concentration or seawater intrusion^121,136^. The observed deterioration trends correspond with the heightened sodium levels recorded (49.16–78.64%) and EC values ranging from 330 to 7010 µS cm^−1^. Collectively, these factors signify a considerable risk of both salinity and sodicity in the irrigated soils of the region. Elevated Na% leads to the dispersion of clay particles, diminishes aggregate stability, and impairs soil permeability; concurrently, high EC exacerbates osmotic stress, complicating water uptake for plants^122,123,143^.

In recent comparable studies, particularly in the semi-arid aquifers of western India, it was observed that when EC surpassed approximately 5000 µS cm^−1^ and Na% exceeded around 60%, over 70% of the samples were classified as doubtful to unsuitable, which is consistent with my findings^123^. Likewise, in the coastal regions of Egypt, Fadl, ElFadl^77^ identified a significant correlation between increasing EC and Na%, and the expansion of areas categorized as unsuitable in Wilcox diagrams. Therefore, the findings from my study area are in agreement with these trends, although they indicate a somewhat more favorable quality of water in the excellent to good and good to permissible categories, implying that certain wells are less affected. In summary, while some wells provide water of relatively good quality, approximately one-third of the samples present a risk that necessitates mitigation.

### IWQI evaluation

The Irrigation Water Quality Index (IWQI), as elucidated by Al-Aizari, Aslaou^104^, is a framework that predominantly integrates a limited number of essential parameters, including but not limited to electrical conductivity (EC), Sodium Adsorption Ratio (SAR), Sodium ions (Na⁺), and Chloride ions (Cl⁻), which are recognized for their significant and direct repercussions on both soil quality and agricultural yield.

These factors play a crucial role in determining key concerns such as salinity, sodicity, and potential toxicity, thereby establishing a clear yet dependable methodology for assessing the suitability of groundwater resources for irrigation purposes. The IWQI serves an invaluable function in facilitating water management-related decision-making processes, as it simplifies the intricacies associated with analyzing vast ranges of data by amalgamating various chemical indicators into a singular numerical value, as supported by the findings of Etikala, Vangala^144^. Recent applications of this analytical technique within the Doukkala region of Morocco have revealed a rather alarming statistic, indicating that approximately 34.02% of groundwater samples fell within the high restriction category, while an additional 22.7% were classified as belonging to the severe restriction class, thus underscoring the escalating challenges posed by sodium and salinity hazards, particularly in regions characterized by intensive agricultural practices, as noted by Al-Aizari, Aslaou^104^.

In a parallel investigation, IWQI computations in the Wadi Fatimah region of Saudi Arabia revealed that despite the fact that nearly 88% of groundwater samples were deemed reasonably suitable for agricultural applications, a considerable portion of these samples nevertheless exhibited levels of restriction that ranged from high to severe due to elevated sodium hazard indices (SAR) and Electrical Conductivity (EC) measurements. This observation reiterates the complexity of the situation, as IWQI calculations in Wadi Fatimah, Saudi Arabia, illustrated that while around 88% of groundwater samples were classified as fairly appropriate for agricultural use, a significant segment of these samples continued to exhibit high to severe restriction levels, primarily attributable to increased sodium hazard indices (SAR) and Electrical Conductivity (EC) parameters, as elaborated by Niyazi, Rajmohan^145^.

Furthermore, according to the IWQI assessments conducted in Makkah Al-Mukarramah, it was determined that approximately 45.5% of the wells were categorized as experiencing “high to severe restriction” concerning their water quality, whereas the remaining wells were classified as having moderate to no restrictions, an indication that poses long-term risks to both crop productivity and soil permeability, as highlighted by Niyazi, Rajmohan^145^. Lakhdari, Bouselsal^137^ investigated a field evaluation of Adrar, located in an arid desert environment in southwest Algeria for its water supply. Where the outcome for irrigation, 31.25% of wells provide good-quality water, while 68.75% are classified as doubtful. Most of the analyzed waters are corrosive, with a tendency toward scaling and CaCO_3_ precipitation.

Conversely, a study executed in Naama, Algeria, uncovered that 42.18% of groundwater samples were categorized as having outstanding quality; however, a notable portion of these samples remained under various restriction categories, predominantly due to elevated concentrations of sodium ions (Na⁺) and chloride ions (Cl⁻), as reported by Hussein, Derdour^128^. Furthermore, an investigation entitled “Tracking groundwater quality changes in Wadi El-Assiuti, Egypt” revealed that IWQI values have shown a concerning upward trend in recent times, signifying a deterioration in water quality that corresponds with rising levels of sodium percentages, SAR, and other pertinent indices, thereby indicating similar pressures are being experienced within the study area, as detailed by Fadl, ElFadl^77^.

**Fig. 8:**
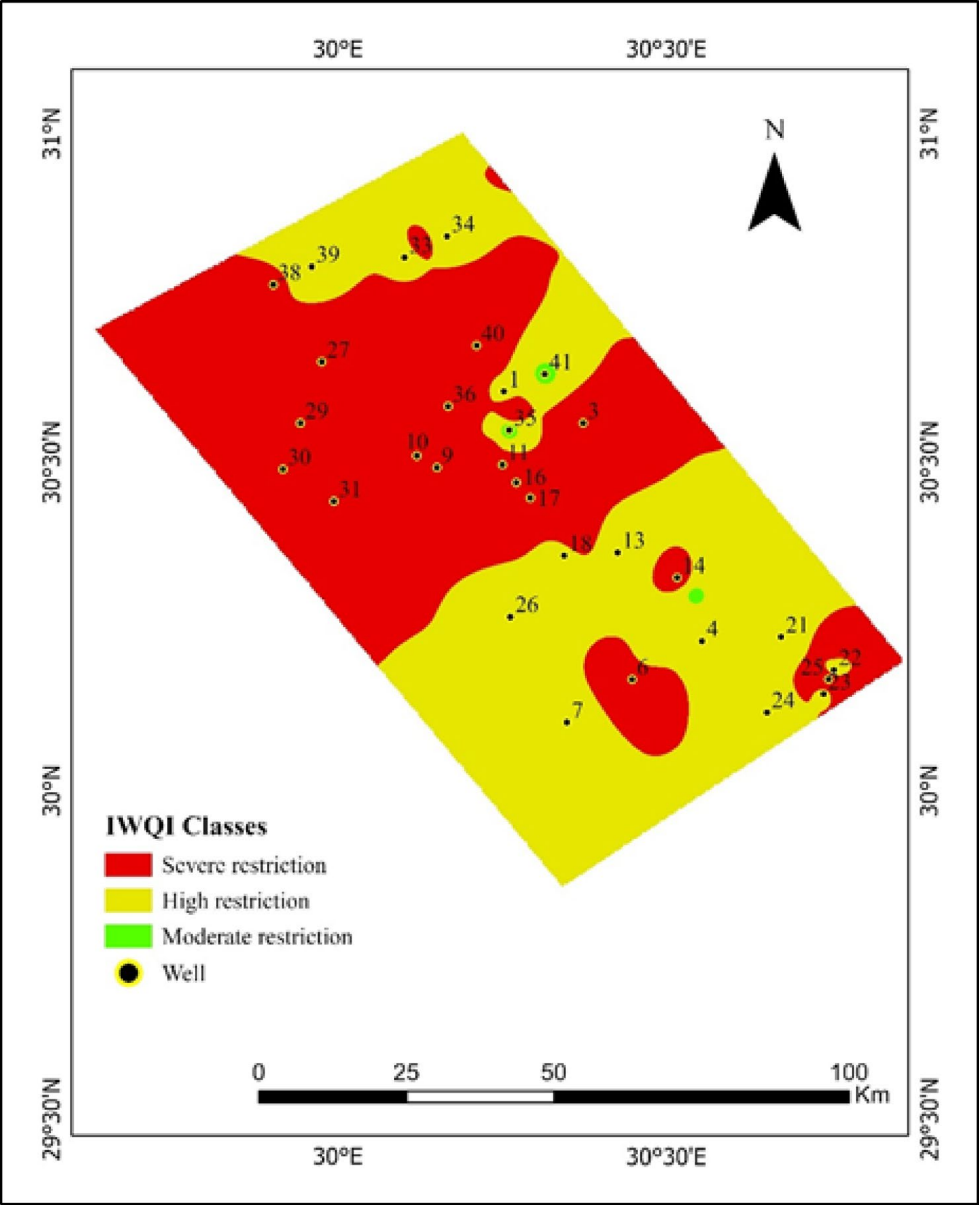
Spatial distribution and classification of the irrigation water quality index (IWQI) in the study area.

The findings from these various studies illustrate that IWQI values exhibited a range spanning from 23.17 to 66.19, with the average value resting at approximately 38.09. This information is further illustrated through the spatial distribution map (Fig. 8), which reveals that a staggering 51.35% of the studied area, covering approximately 3844.31 km², has been classified under severe restriction, while 48.41% of the area, amounting to around 3624.51 km², falls within the high restriction category, and a mere 0.24% of the area, equivalent to 17.96 km², is categorized as experiencing moderate restriction, as presented in Table 9. When considering the aforementioned data, it becomes evident that 7.32% of the irrigation water samples are categorized under moderate restriction, while 34.15% fall under high restriction, and a striking 58.54% are classified under severe restriction.

**Table 9:** Classification of the irrigation water quality index (IWQI) with its corresponding spatial coverage in the study area.

Index	Class	Area (Km^2^)	Area (%)
IWQI	Severe restriction	3844.31	51.35
	High restriction	3624.51	48.41
	Moderate restriction	17.96	0.24

Notably, when comparing the samples obtained from the research area with those from the previously mentioned studies conducted in Morocco, Saudi Arabia, and Egypt, it becomes apparent that there exists a disproportionately higher prevalence of samples classified within the severely restricted categories in this particular study. This indicates that the presence of salt, sodium, or other ionic risks significantly impacts the water resources within the studied region, potentially due to factors such as local geology, agricultural practices, or insufficient drainage. Consequently, it is essential to implement certain strategies, including the mixing of water with purer sources, the cultivation of salt-tolerant crops, the enhancement of drainage systems, the improvement of soil structure (for instance, through the application of gypsum or organic materials), and the establishment of consistent monitoring programs for Irrigation Water Quality Index (IWQI) and its contributing factors, all of which should be prioritized to ensure sustainable irrigation.

### GIS-based spatial modeling and zoning of groundwater suitability for irrigation management

**Fig. 9:**
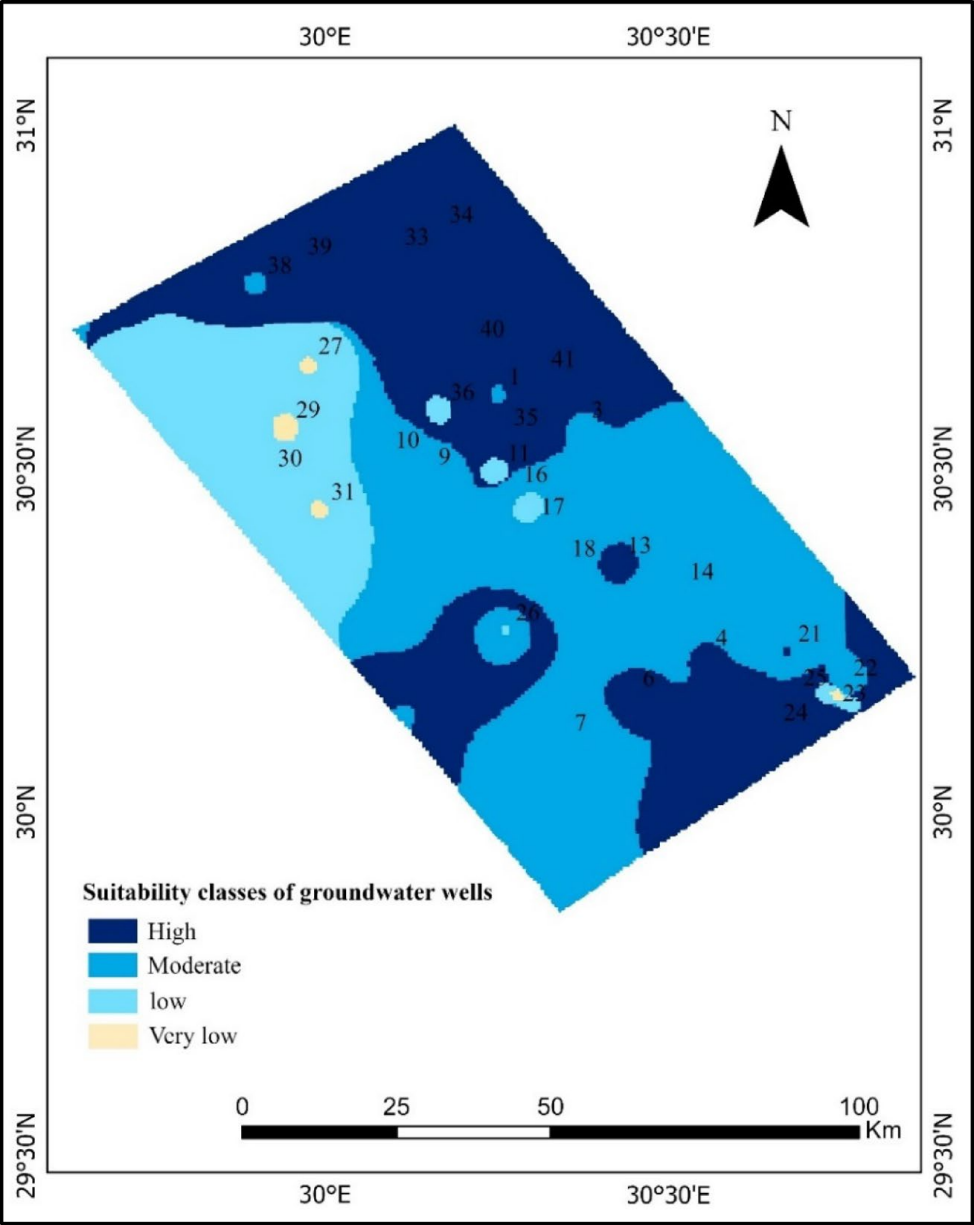
Classification map of groundwater wells according to irrigation suitability (High – Moderate –Low – Very low).

The geographical study area has been systematically categorized into four distinct groundwater-suitability classifications based on a thorough spatial integration analysis, as illustrated in Fig. 9 and summarized in Table 10, which delineates the following classifications: High (3,012.15 km², accounting for 42.42% of the total area), Moderate (2,908.87 km², representing 40.97%), Low (1,153.10 km², or 16.24%), and Very Low (26.23 km², which constitutes 0.37%). The boundaries of these classifications are indicative of the combined influence of several significant hydrochemical drivers, including parameters such as Potential Irrigation (PI), Residual Sodium Carbonate (RSC), Sodium Adsorption Ratio (SAR), Kelly’s Ratio (KR), percentage of Sodium (Na%), and Total Dissolved Solids (TDS), alongside their geographical gradients, a process that has been effectively accomplished through the GIS weighted-overlay workflow.

**Table 10:** Distribution of groundwater suitability classes (High–Moderate–Low–Very low) with their corresponding areas and percentages.

Suitability classes of groundwater wells	Area (Km^2^)	Area%
High	3012.15	42.42
Moderate	2908.87	40.97
Low	1153.10	16.24
Very low	26.23	0.37

From a scientific viewpoint, the majority of groundwater samples collected within the designated study area exhibit ionic compositions and electrical conductivities that remain well within the acceptable limits for irrigation, particularly when managed under optimal agricultural practices, which is substantiated by the substantial predominance of High and Moderate suitability zones that collectively encompass approximately 83% of the total surveyed area. However, it is essential to note that there exist notable regions characterized by low suitability (approximately 16%) and even smaller areas classified as very low, which serve to indicate the presence of localized processes that have the potential to elevate the risk associated with irrigation practices. These detrimental processes are primarily attributed to salinity issues, evidenced by elevated electrical conductivity (EC) and total dissolved solids (TDS), as well as concerns regarding sodium hazards, reflected in high percentages of sodium (Na%) and sodium adsorption ratio (SAR), which together significantly diminish the agronomic suitability of the water for irrigation purposes in two critical ways: firstly, by increasing the osmotic potential of the soil, thereby reducing the availability of water to plants, and secondly, by prompting the dispersion of clay particles and subsequently diminishing soil permeability when sodium ions (Na⁺) prevail as the dominant exchange cation. Recent academic investigations focused on groundwater irrigation have elucidated and clarified the various factors that govern groundwater quality, providing valuable insights into the underlying mechanisms at play, as noted by Eid, Elbagory^146^.

However, it is essential to note that there exist notable regions characterized by low suitability (approximately 16%) and even smaller areas classified as very low, which serve to indicate the presence of localized processes that have the potential to elevate the risk associated with irrigation practices. These detrimental processes are primarily attributed to salinity issues, evidenced by elevated electrical conductivity (EC) and total dissolved solids (TDS), as well as concerns regarding sodium hazards, reflected in high percentages of sodium (Na%) and sodium adsorption ratio (SAR), which together significantly diminish the agronomic suitability of the water for irrigation purposes in two critical ways: firstly, by increasing the osmotic potential of the soil, thereby reducing the availability of water to plants, and secondly, by prompting the dispersion of clay particles and subsequently diminishing soil permeability when sodium ions (Na⁺) prevail as the dominant exchange cation. Recent academic investigations focused on groundwater irrigation have elucidated and clarified the various factors that govern groundwater quality, providing valuable insights into the underlying mechanisms at play, as noted by Eid, Elbagory^146^.

The discerned spatial pattern, characterized by clusters of lower suitability classifications, aligns with established and well-documented causal pathways that are commonly observed in arid and semi-arid aquifers: these include, firstly, anthropogenic inputs such as return flows originating from irrigation activities and inflows resulting from the use of fertilized or saline irrigation practices, which are known to contribute to localized increases in concentrations of sodium ions (Na⁺) and chloride ions (Cl⁻); secondly, the effects of evaporative concentration coupled with the impacts associated with shallow water-table depths that lead to heightened levels of electrical conductivity (EC) and conservative ions; and thirdly, the mixing of groundwater with older, more mineralized sources along preferential flow paths that exacerbate salinity issues. Given that salinity and sodicity factors are incorporated into the higher scores during the index calculations, it is evident that the Irrigation Water Quality Index (IWQI) is experiencing a decline in regions where these problematic processes are prevalent. This interpretation is further substantiated by comprehensive field investigations that have employed hydrochemical source apportionment techniques combined with IWQI mapping methodologies, which have consistently documented a similar correlation between elevated electrical conductivity (EC) and sodium concentrations (Na) indicative of salinization processes, and the occurrence of low IWQI zones, as illustrated in the findings presented by Singh, Singh^147^.

From a managerial perspective, the zoning framework established by this model delineates specific responsibilities that ought to be undertaken by each identified class: For instance, in high suitability zones, which exhibit optimal conditions, it is anticipated that these areas will be capable of sustaining a majority of irrigated agricultural crops; Conversely, in moderate suitability zones, there exists a pressing necessity for regular salinity assessments, the implementation of water-saving irrigation techniques aimed at mitigating salt accumulation, and the careful selection of crop cultivars that possess the ability to endure moderate levels of salinity; In contrast, the low and very low suitability zones require immediate remedial strategies, which may encompass the blending of irrigation water with sources that possess a lower salinity profile, the enhancement of drainage and leaching practices to effectively manage long-term salinity levels, the application of gypsum in areas where sodicity is a predominant concern, and the implementation of measures to halt further salinity infiltration. These strategic recommendations are in alignment with contemporary guidelines and established best practices that are acknowledged globally for agricultural practices in arid regions, as highlighted by the research conducted by Anyango et al. in 2024.

Ultimately, it is imperative to underscore the critical importance of addressing uncertainty and maintaining vigilant observation throughout this process. Prior to making any irrevocable commitments to long-term irrigation initiatives within marginally suitable zones, it is highly advisable to employ longitudinal resampling methodologies and, where feasible, to conduct temporal analyses or adopt probabilistic approaches—such as ensemble Irrigation Water Quality Index (IWQI) scenarios or geostatistical cross-validation techniques—to rigorously quantify and interpret the trends and uncertainties associated with the data.

From a management standpoint, the zoning designates distinct tasks that each class should take: With consistent observation, high zones may sustain the majority of irrigated crops; Regular salinity monitoring, water-saving irrigation to prevent salt buildup, and crop selection for cultivars that can withstand moderate salt levels are all necessary in moderate zones; Remedial measures are required in low/very low zones. These include blending with sources of lower salinity, improving drainage and leaching to control long-term salinity buildup, using gypsum where sodicity is predominant, and stopping salinity intake. These ideas align with current guidelines and global best practices for arid agricultural areas^53^.In the end, it is important to stress the necessity of uncertainty and observation. Before committing to long-term irrigation development in marginal zones, it is advised to use longitudinal resampling and, if practical, temporal analyses or probabilistic approaches (such as ensemble IWQI scenarios or geostatistical cross-validation) to quantify and interpret trend and uncertainty.

The main objective of the study was to monitor the state of groundwater quality in the New Delta region and its impact on the sustainability of this resource under increasing consumption and slow recharge processes, the main source of which is the Nile River, with seawater intrusion into the groundwater, which negatively affects groundwater quality and may be considered a threat to the availability of this resource for future generations. Therefore, the study recommends continuous and better annual monitoring of groundwater levels and quality in these areas. The state has established the EL-Hammam wastewater treatment station, which is the largest agricultural drainage water treatment plant, where treatment is carried out to the third degree to compensate for the shortage of groundwater resources in the New Delta. However, the study recommends increasing the treatment degree to the fourth degree to increase the quality of the treated water so that a portion of it can be used to recharge the groundwater aquifer, in addition to the small amount that comes through rainfall in this region to compensate for the shortage, in order to achieve sustainability in water resource management in this region.

### Cluster dendrogram and heatmap

**Fig. 10:**
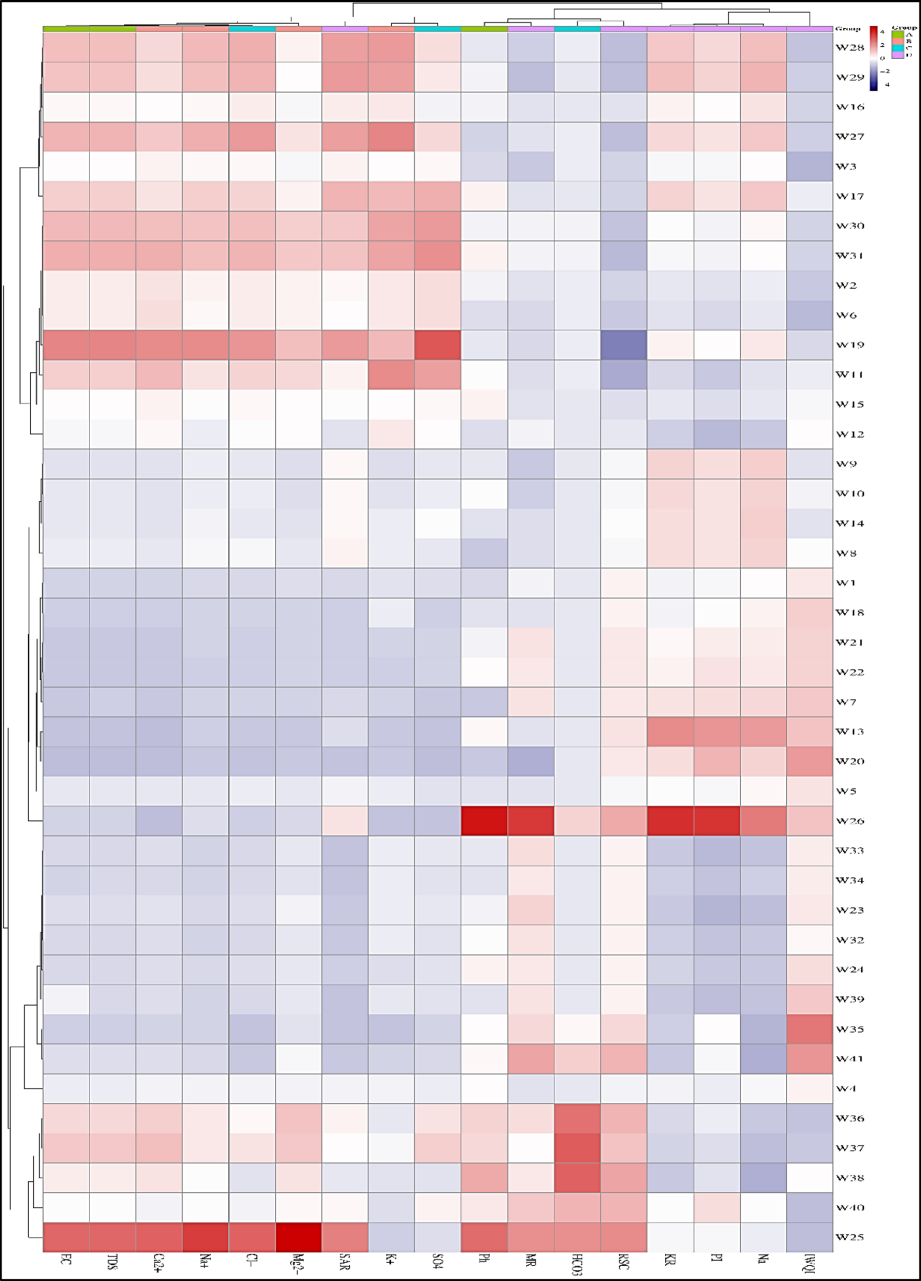
Cluster Heatmap correlation analysis between the groundwater physical parameters, chemical parameters, and irrigation water quality indices for the different studied wells. *SAR* Sodium adsorption ratio, *MR* Magnesium adsorption ratio, *Na* Percentage of sodium, *PI* Permeability index, *RSC* Residual sodium carbonate, *KR* Kelly ratio, *IWQI* irrigation water quality index.

The bi-directional cluster analysis and the resulting Dendrogram derived from various water physical parameters, chemical parameters, and irrigation water quality indices (IWQI), where the two-way cluster analysis and the produced Dendrogram based on the 17 measurements distinctly grouped them into four categories (A, B, C, and D) (Fig. 10). In line with this, each of the four categories (A, B, C, and D) is further divided into subcategories, each consisting of one of the following: group A divided into three subcategories (water physical parameters: pH, EC, and TDS), in the same context the group B divided into four subcategories(water chemical cations: Ca^2+^, Mg^2+^, Na^+^, and K^+^), on the other hand group C divided into three subcategories(water chemical anions: $\:{HCO}_{3}^{-}$, Cl-, and $\:{SO}_{4}^{2-}$), while the last group D divided into seven subcategories(water quality indices: SAR, RSC, Na%, KR, MR, PI, and IWQI).

There was no consistent trend in the effect of water analyses for each of the study wells on the quality of irrigation water. Rather, the effects varied between positive and negative under the same well and under the same used indicator, which made it impossible to reach which of them had a greater effect on the quality of irrigation water in terms of the wells or in terms of the evaluation criteria due to the large overlap between the study indicators and the diversity of their effects. To avoid this, we resorted to analysis using Pearson’s Correlation so that we could determine which criteria and indicators had a positive or negative effect and to what degree on the irrigation water quality index.

**Fig. 11:**
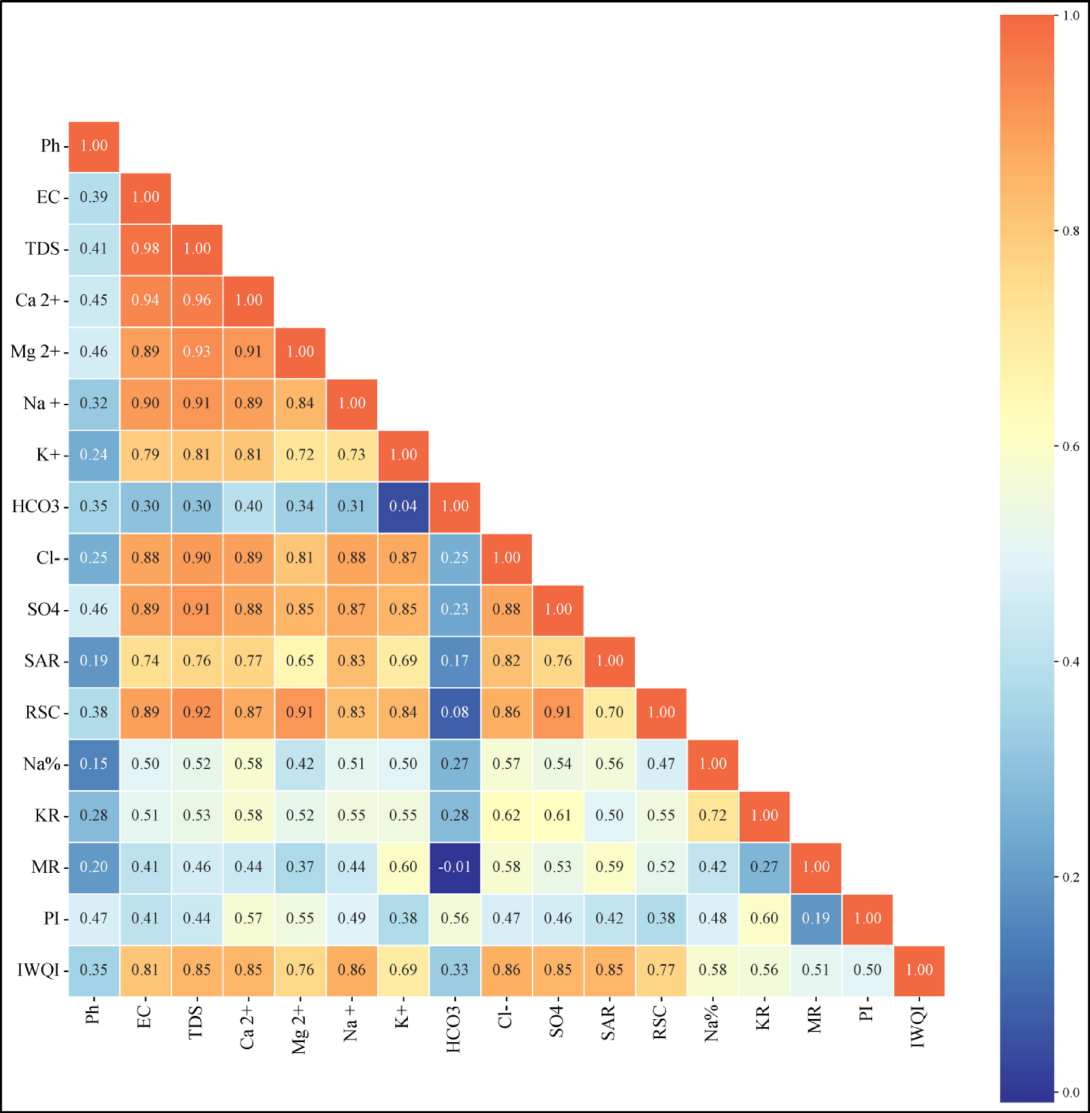
Correlation analysis between groundwater physical parameters, chemical parameters, and indices. *SAR* Sodium adsorption ratio, *MR* Magnesium adsorption ratio, *Na* Percentage of sodium, *PI* Permeability index, RSC Residual sodium carbonate, *KR* Kelly ratio, *IWQI* irrigation water quality index.

Likewise, as shown in (Fig. 11), Pearson’s correlation analysis was employed to determine the positive and negative relationships among the parameters and indices being studied. In a correlation Heatmap, values usually range from − 1 to + 1 (Pearson correlation coefficient. A correlation Heatmap is not just a visual tool; it adds clear scientific value by quantifying and summarizing relationships among variables.

The Pearson’s correlation Heatmap shows how strongly groundwater parameters vary together, which helps to detect common geochemical origins, and infer shared controlling processes. The findings from the Pearson’s correlation analysis revealed a strong positive association between irrigation water quality index (IWQI) and the following parameters and indices: Cl^−^ (0.86), Na^+^ (0.86), $\:{SO}_{4}^{2-}$(0.85), TDS (0.85), Ca^2+^ (0.85), SAR (0.85), EC (0.81), RSC (0.77), Mg^2+^ (0.76), K^+^ (0.69), on the other hand these parameters and indices have the lowest positive correlation with IWQI as follows: Na% (0.58), KR (0.56), MR (0.51), PI (0.50), pH (35), and $\:{HCO}_{3}^{-}$ (0.33). These correlations imply that Na and Cl concentrations rise with increasing total ionic load (as indicated by EC), which is probably caused by evaporative concentration and mineral dissolution. These patterns have also been documented in groundwater studies in West EL-Minia, Egypt (strong correlations among EC, Na⁺, Cl⁻, Mg²⁺, Ca²⁺) and Quaternary aquifers of the Nile Delta under the influence of seawater intrusion (strong EC- Na⁺ - Cl⁻ associations)^121,148^.

### Principal component analysis (PCA)

**Fig. 12:**
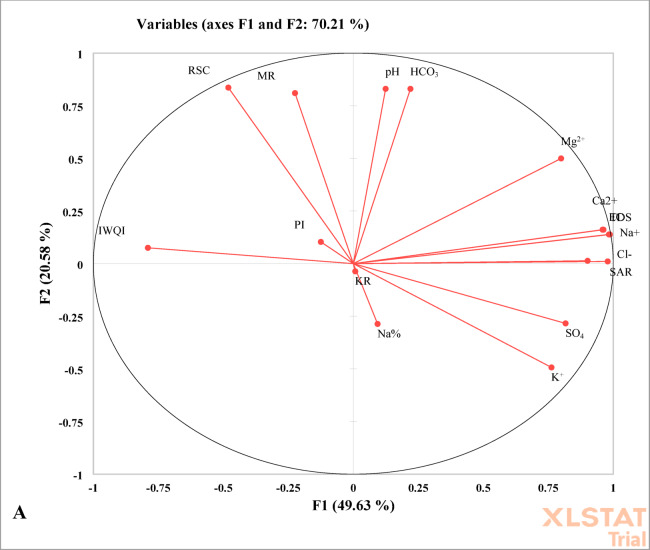

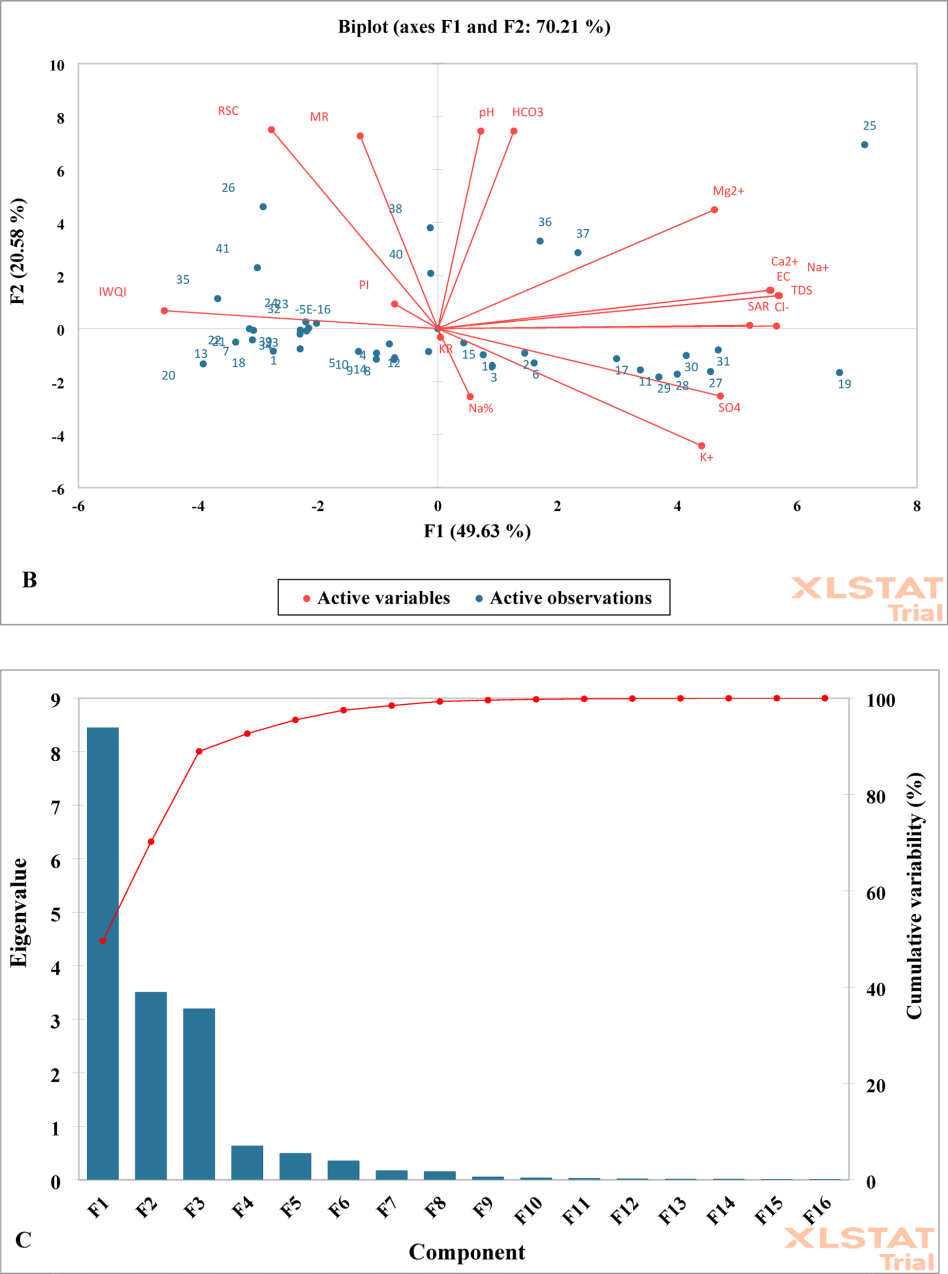
The Principal Component Groundwater quality parameter analysis: (**A**) correlation circle displaying the connections between irrigation indices and hydrochemical variables, (**B**) biplot displaying the connections between active observation and active variables, and (**C**) screen plot displaying the variance accounted for by each component.

The PCA outcomes (Fig. 12 A) indicated that the first two principal components (F1 and F2) represented 70.21% of the total variance (49.63% and 20.58%, respectively), underscoring that a limited number of dominant geochemical processes account for the majority of variability in groundwater chemistry (Fig. 12A,B). The first axis (F1) exhibited a strong correlation with salinity-related parameters (EC, TDS, Cl⁻, SO₄²⁻, Na⁺, SAR, and Na%), suggesting the effects of evaporation, irrigation return flow, and localized seawater intrusion, whereas the second axis (F2) was linked to alkalinity-related variables (HCO₃⁻, RSC, MR, and pH), which reflect the processes of carbonate dissolution and cation exchange. Indices such as KR and PI demonstrated moderate loadings, indicating their partial dependence on Na⁺ in relation to Ca²⁺ and Mg²⁺.

The scree plot (Fig. 12C) validated that only the first two components are statistically significant, with the following components accounting for negligible variance. The result of the Kaiser-Meyer-Olkin (KMO) test was 0.693, and the Bartlett’s sphericity test indicates that the value is lower than 0.001, which indicates that the sample adequacy for factor analysis is acceptable. In their groundwater assessment of the Quaternary aquifer in Egypt’s southwest Nile Delta^1^ reported a similar finding, with a KMO value of 0.615, which was deemed sufficient for the study. For instance, comparable KMO values were utilized in statistical evaluations in the Nile Delta and coastal aquifers, where PCA extracted significant components, indicating that the data structure supports multivariate methods, albeit cautiously^121,149^.These findings align with multivariate studies from other semi-arid aquifers. For instance, In Lioua Plain, Algeria, PCA identified three components that explained approximately 85% of the variance, primarily due to evaporation, water-rock interaction, and mineral dissolution^150^. Similarly, in Asyut, Egypt, three rotated components explained approximately 74% of the variance, and were associated with both natural weathering and human input^149^. These results are consistent with numerous recent hydrochemical assessments.

Seasonal impacts seem to be significant: whereas salt and chloride concentrations grow in dry seasons due to evaporative concentration, bicarbonate levels are relatively greater during wet periods, perhaps because of increased carbonate dissolution with recharge. This seasonal pattern is consistent with other studies that have found that EC, Na⁺, and Cl⁻ increase during the dry period in the reclaimed agricultural regions, similar patterns of Cl⁻-Na⁺ correlation and elevation during the dry season were reported^124,151–153^.

## Conclusions

Groundwater in Egypt’s New Delta region is generally suitable for irrigation under current reclamation and agricultural expansion activities. Most samples comply with international irrigation standards and are classified as suitable to good according to IWQI and related indices. However, sodium-related hazards emerge as the principal constraint to sustainable irrigation, as elevated Na⁺ and Cl⁻ concentrations in localized zones pose significant salinity and sodicity risks, particularly in newly reclaimed and fine-textured soils.

Supporting hydrochemical and spatial analyses indicate that pH, Ca²⁺, Mg²⁺, and SO₄²⁻ levels largely fall within permissible limits, suggesting minimal risk from these parameters. Spatial mapping of SAR, Na%, RSC, PI, MR, KR, and IWQI demonstrates that unsuitable conditions are geographically restricted and strongly associated with high sodium and chloride contents. Principal Component Analysis further confirms that groundwater chemistry is predominantly controlled by salinity and sodicity factors (EC, TDS, Na⁺, Cl⁻, and SAR), while carbonate equilibria, water–rock interactions, evaporation, and agricultural return flows exert secondary influences.

These findings highlight the need for proactive, site-specific management strategies to ensure long-term irrigation sustainability in the New Delta. Recommended measures include blending groundwater with lower-salinity sources, applying soil amendments such as gypsum, adopting salt-tolerant crops, optimizing irrigation scheduling, and continuously monitoring Na⁺ and Cl⁻ hotspots. Overall, while groundwater resources in the New Delta are broadly suitable for irrigation, adaptive salinity management is essential to support resilient agricultural development and inform effective irrigation planning and policy decisions.

## Data Availability

The researchers provided the experimental requirements of raw materials and system design through the Science, Academy of Scientific Research and Technology (ASRT) fund, while the data from previous studies and research was obtained through the Cairo University platform, which provides research regularly, all data are available at request.

## Code availability

(Software application or custom code): All codes will be available at request.
